# Chitosan-supported CuI-catalyzed cascade reaction of 2-halobenzoic acids and amidines for the synthesis of quinazolinones

**DOI:** 10.3762/bjoc.21.67

**Published:** 2025-04-28

**Authors:** Xuhong Zhao, Weishuang Li, Mengli Yang, Bojie Li, Yaoyao Zhang, Lizhen Huang, Lei Zhu

**Affiliations:** 1 School of Chemistry and Materials Science, Hubei Provincial Engineering Research Center of Key Technologies in Modern Paper and Hygiene Products Manufacturing, Hubei Engineering University, Xiaogan 432000, China

**Keywords:** chitosan-supported CuI catalyst, cyclization reaction, mild conditions, quinazolinone

## Abstract

A chitosan-supported CuI (CS@CuI) catalyst was developed for the synthesis of quinazolinones from 2-halobenzoic acids (including iodine and bromine) and amidines. The reaction proceeds under mild reaction conditions, demonstrating a broad substrate scope (30 examples) and good catalytic efficiency (up to 99% yield).

## Introduction

**Scheme 1:**
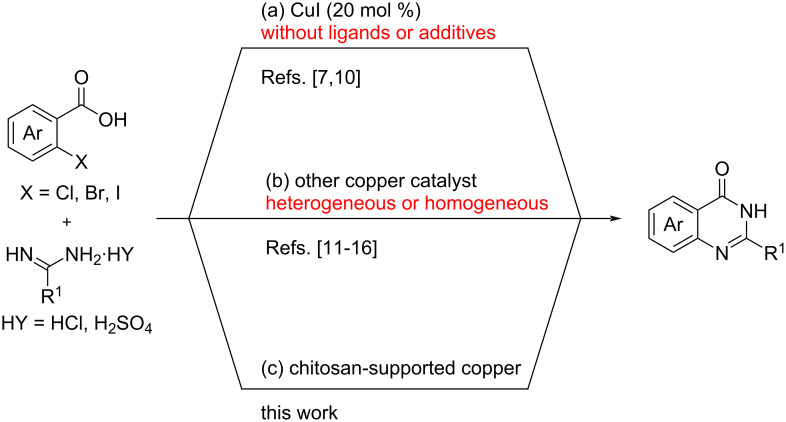
Copper-catalyzed cascade reaction of 2-halobenzoic acids and amidines for the synthesis of quinazolinones.

Quinazolinones are not only a key core of nitrogen-containing benzo heterocyclic compounds found in many natural products and bioactive molecules [1–3], but can also be readily converted into other functional compounds under specific conditions [4–5]. Due to their significant biological relevance and potential applications, numerous synthetic methods have been recently developed to synthesize these useful intermediates [6–9]. Among these methods, the cascade reaction between *ortho*-halogen (e.g., chlorine, bromine or iodine) substituted benzoic acids and amidines has become a prominent route to synthesize the corresponding quinazolinones [10–18]. In 2009, Fu and co-workers found that copper(I) could effectively promote this cascade reaction for the synthesis of quinazolinones without the need for additional ligands or additives (Scheme 1a) [7,10]. Since then, various copper-based catalysts, both homogeneous and heterogeneous, have been explored (Scheme 1b) [11–16]. For example, Wang’s group developed a magnetically recoverable and reusable Fe_3_O_4_ nanoparticle-supported copper(I) catalyst with excellent catalytic efficiency for quinazolinone synthesis [11]. In addition, Cai et al. reported that MCM-41-immobilized tridentate nitrogen-supported copper(I) [MCM-41-3N–CuI] served as a highly efficient, reusable heterogeneous catalyst for this cascade reaction, achieving good to excellent yields without any loss of activity even after ten cycles of simple filtration-based recovery [12]. Moreover, a copper catalyst has been shown to function effectively in both organic and aqueous media [13–14]. Furthermore, dicopper(I) complexes can also be used as an effective catalyst in Ullmann-type *N*-arylation/cyclization of 2-bromobenzoic acids with amidines, providing the corresponding quinazolinones in good yields [15]. Despite the high efficiency of the above-mentioned copper catalysts in the synthesis of quinazolinones, and the wide application of the chitosan-supported copper catalyst in various organic transformations [19–21], the use of chitosan-supported copper for quinazolinone synthesis has not been reported. As part of our ongoing research interest in chitosan and chitosan-supported copper catalysts in organic transformations [22–24], we intended to investigate the use of chitosan-supported copper as a catalyst for the synthesis of quinazolinones from 2-halobenzoic acids and amidines under mild reaction conditions (Scheme 1c).

## Results and Discussion

The initial reactions commenced with 2-iodobenzoic acid (**1a**, 0.5 mmol, 1.0 equiv) and acetamidine hydrochloride (**2a**, 0.75 mmol, 1.5 equiv) as model substrates, Na_2_CO_3_ (1.25 mmol, 2.5 equiv) as a base, and chitosan-supported copper (5.0 mol %) as the catalyst under an argon atmosphere (Table 1). First, various solvents were investigated. When nonprotonated solvents such as THF and toluene were used, the yields were relatively low (Table 1, entries 1 and 2, 39 and 27% yields), indicating poor catalytic activity in these solvents. In contrast, using proton solvents (MeOH, iPrOH and H_2_O) led to improved yields (Table 1, entries 3−5, 51−60% yields). Notably, the reaction was also successful in water, affording the target product in moderate yield (Table 1, entry 5, 51% yield). Next, to further improve the yield, a mixed solvent of iPrOH and H_2_O was examined. The reaction conducted with a solvent ratio of iPrOH/H_2_O = 4:1 gave an 83% yield (Table 1, entry 6), while a ratio of iPrOH/H_2_O = 9:1 resulted in an 89% yield (Table 1, entry 7). In the optimal solvent (iPrOH/H_2_O = 9:1), other chitosan-supported copper catalysts, such as chitosan-supported on CuBr (CS@CuBr), chitosan-supported on Cu(OAc)_2_ (CS@Cu(OAc)_2_), chitosan-supported on Cu(acac)_2_ (CS@Cu(acac)_2_) and chitosan-supported on CuSO_4_ (CS@CuSO_4_) were explored, and the results showed that CS@CuI was the most effective catalyst (Table 1, entries 7−11, 65−89% yields). To further enhance the reaction yield, the reaction temperature was increased to 90 °C, and the target product **3a** was obtained in 96% isolated yield (Table 1, entry 12). Control experiments indicated poor results when no catalyst was used, with the corresponding product obtained only in 31% yield (Table 1, entry 13). When CuI or chitosan alone was used as a catalyst, the reaction occurred but with less efficiency (Table 1, entries 14 and 15, 80 and 40% yields). In addition, when the reaction time was reduced, the yields decreased accordingly (Table 1, entries 16−18, 70−94% yields). Finally, when the reaction was carried out under open air, the catalytic activity decreased and only 45% yield of the target product was obtained (Table 1, entry 19).

**Table 1:** Optimization of reaction conditions^a^.

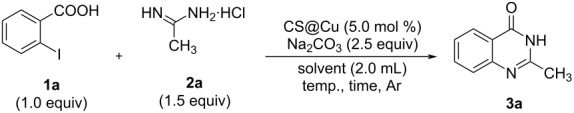					

Entry	CS@Cu	Solvent	Temp. (°C)	Time (h)	Yield (%)^b^

1	CS@CuI	THF	80	12	39
2	CS@CuI	toluene	80	12	27
3	CS@CuI	MeOH	80	12	55
4	CS@CuI	iPrOH	80	12	60
5	CS@CuI	H_2_O	80	12	51
6	CS@CuI	iPrOH/H_2_O (4:1)	80	12	83
7	CS@CuI	iPrOH/H_2_O (9:1)	80	12	89
8	CS@CuBr	iPrOH/H_2_O (9:1)	80	12	87
9	CS@Cu(OAc)_2_	iPrOH/H_2_O (9:1)	80	12	65
10	CS@Cu(acac)_2_	iPrOH/H_2_O (9:1)	80	12	65
11	CS@CuSO_4_	iPrOH/H_2_O (9:1)	80	12	67
**12**	**CS@CuI**	**iPrOH/H** **_2_** **O (9:1)**	**90**	**12**	**99 (96)** **^c^**
13	–	iPrOH/H_2_O (9:1)	90	12	31
14	CuI	iPrOH/H_2_O (9:1)	90	12	80
15	CS	iPrOH/H_2_O (9:1)	90	12	40
16	CS@CuI	iPrOH/H_2_O (9:1)	90	8	94
17	CS@CuI	iPrOH/H_2_O (9:1)	90	5	83
18	CS@CuI	iPrOH/H_2_O (9:1)	90	3	70
19^d^	CS@CuI	iPrOH/H_2_O (9:1)	90	12	45

^a^Reaction conditions: **1a** (0.5 mmol, 1.0 equiv), acetamidine hydrochloride **2a** (0.75 mmol, 1.5 equiv), CS@Cu (5.0 mol %), Na_2_CO_3_ (1.25 mmol, 2.5 equiv), solvent (2.0 mL) at argon atmosphere. ^b^The yield was determined by ^1^H NMR analysis with dibromomethane as an internal standard. ^c^Isolated yield in parentheses. ^d^The reaction was performed under open air.

**Scheme 2:**
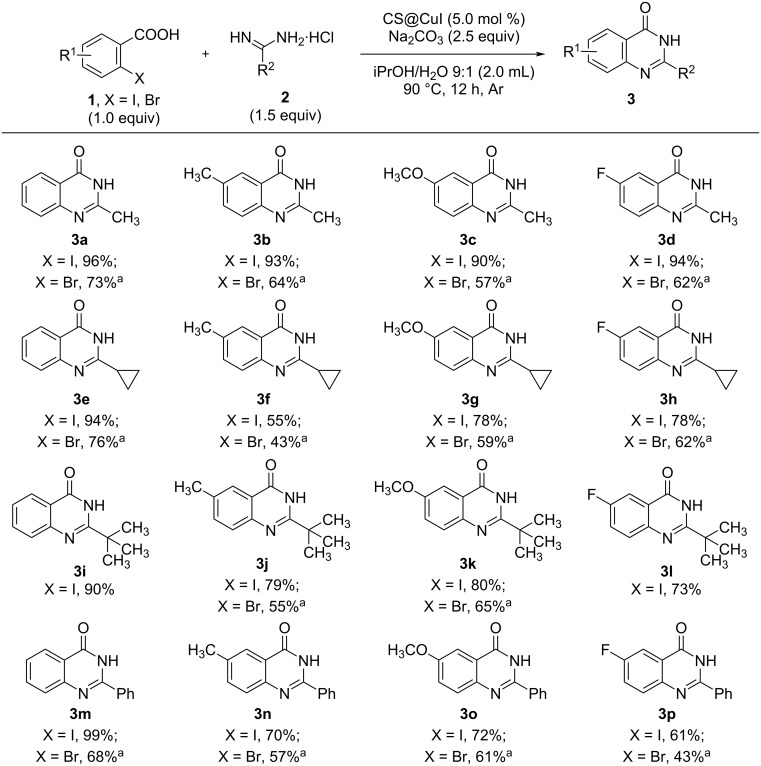
Substrate scope. Reaction conditions: **1** (0.5 mmol, 1.0 equiv), amidines hydrochloride **2** (0.75 mmol, 1.5 equiv), CS@CuI (10.0 mg, ICP: 14.6%, 5.0 mol %), Na_2_CO_3_ (1.25 mmol, 2.5 equiv), iPrOH/H_2_O 9:1 (2.0 mL), 90 °C, 12 h, argon atmosphere; ^a^**1** (0.2 mmol), amidine hydrochloride **2** (0.3 mmol, 1.5 equiv), CS@CuI (5.0 mol %), Na_2_CO_3_ (1.25 mmol, 2.5 equiv), iPrOH/H_2_O 9:1 (2.0 mL), 90 °C, 12 h, argon atmosphere.

With the optimized conditions in hand, we explored the substrate scope of the CS@CuI-catalyzed cascade reactions of 2-halobenzoic acids (including 2-iodobenzoic acid and 2-bromobenzoic acid) with amidines (Scheme 2). Initially, when the amidine substituent (R^2^) is a methyl group, we investigated the reactions with various substituted 2-halobenzoic acids. The reactivity of 2-iodobenzoic acid derivatives (**3a**–**d**, 90−96% yields) was higher than that of 2-bromobenzoic acid derivatives (**3a**–**d**, 57−73% yields), the electronic properties of the substituents on the benzene ring had little effect on the reactivity. When the amidine substituent (R^2^) was changed to a cyclopropyl group, the yields of all reaction decreased, especially when substituents were present on the benzene ring (**3e**–**h**, 55−94% yields for 2-iodobenzoic acid, 43–76% yields for 2-bromobenzoic acid). We then investigated the reactions of different 2-halobenzoic acid derivatives with amidines where R^2^ was a *tert*-butyl group. The results showed that 2-bromobenzoic acid derivatives (**3j**–**k**, 55−65% yields) displayed lower activity compared to 2-iodobenzoic acid derivatives (**3i**–**l**, 73−90% yields), with a decrease in reaction activity observed when substituents were presented on the benzene ring. Finally, we examined reactions with 2-halobenzoic acid derivatives where the R^2^ substituent was a phenyl group. In this case, the reactivity of 2-iodobenzoic acid derivatives (**3m**–**p**, 61–99% yields) was again superior to that of 2-bromobenzoic acid derivatives (**3m**−**3p**, 43−68% yields). The reactivity of 2-halobenzoic acid without substituents was obviously better than that of substituted derivatives. Overall, these results demonstrate that the reaction has a broad substrate scope, with 2-iodobenzoic acid derivatives showing higher reactivity than 2-bromobenzoic acid derivatives.

**Scheme 3:**
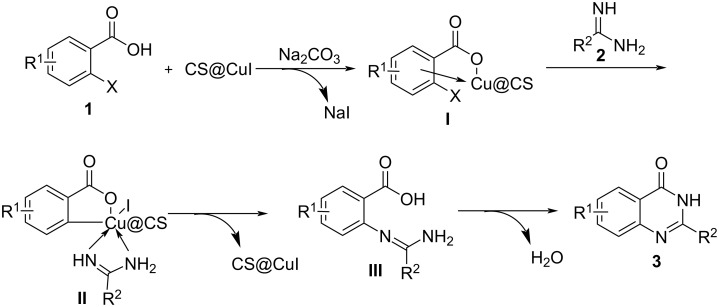
Proposed mechanism for the CS@CuI-catalyzed synthesis of quinazolinones.

Based on previously reported literature [7,13], a mechanism for the copper-catalyzed formation of quinazolinones is proposed in Scheme 3. Initially, the 2-halobenzoic acid **1** coordinates with CS@CuI to form intermediate **I** in the presence of Na_2_CO_3_, which acts as a base. Subsequently, **I** undergoes oxidative addition and complexation with the amidine **2** to generate intermediate **II**. This intermediate then undergoes reductive elimination to form intermediate **III**, releasing CS@CuI back into the system. Finally, the coupling reaction between the carboxyl and amino groups in **III** yields the target quinazolinone **3**.

**Scheme 4:**
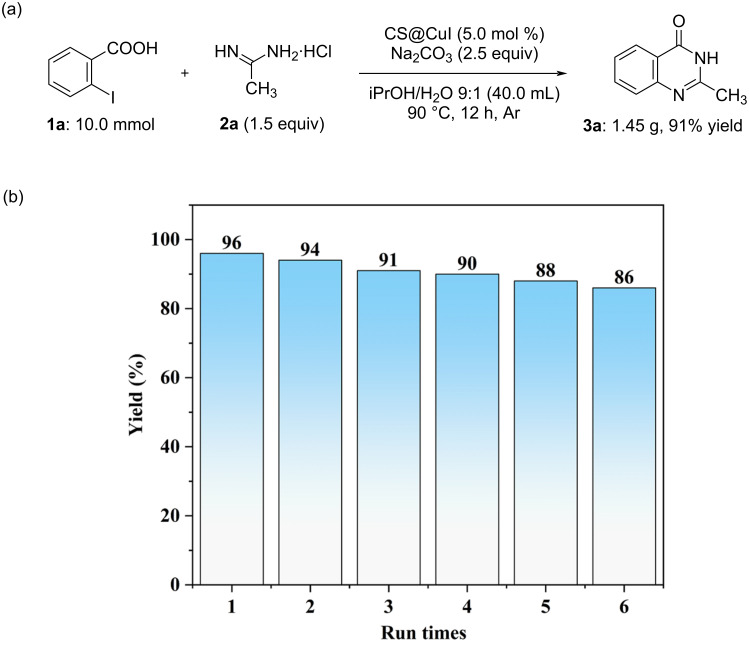
Scaling-up experiment (a) and recyclability of CS@CuI (b).

To demonstrate the practicality of this reaction in organic synthesis, the reaction was scaled up to the gram level. For instance, the desired product **3a** was obtained in 91% yield (1.45 g) when the reaction was conducted on a 10.0 mmol scale under optimized conditions (Scheme 4a). The recyclability of heterogeneous catalysts is a critical factor in assessing their practical utility in transition metal-catalyzed reactions. Therefore, the recyclability of CS@CuI was evaluated in the reaction of 2-iodobenzoic acid (**1a**) with **2a**, as illustrated in Scheme 4b. In each cycle, the recovered CS@CuI was simply centrifuged, filtered, washed, dried, and then reused with fresh substrate under the optimized conditions. The results demonstrate that the catalyst retains good catalytic activity (yields no less than 86%) even after six cycles, and ICP analysis of the filtered aqueous solution after the reaction confirmed no detectable leaching of CuI.

## Conclusion

In summary, we have developed a CS@CuI-catalyzed cascade reaction of 2-halobenzoic acids (including iodine and bromine derivatives) and amidines for the synthesis of quinazolinones. This approach features mild reaction conditions, broad substrate scope (30 examples), and high efficiency (up to 99% yield). In a word, this work presents a novel and efficient protocol for the construction of quinazolinones and offers significant research value.

## Supporting Information

File 1 Full experimental details, characterization data and copies of NMR spectra of all products.

## Data Availability

All data that supports the findings of this study is available in the published article and/or the supporting information of this article.
