# Supplementary material for: Chitosan-supported CuI-catalyzed cascade reaction of 2-halobenzoic acids and amidines for the synthesis of quinazolinones
Source: Beilstein J Org Chem. 2025 Apr 28;21:839–44. doi: 10.3762/bjoc.21.67 (PMC12051478; doi:10.3762/bjoc.21.67)
Supplement: File 1 — Full experimental details, characterization data and copies of NMR spectra of all products. [file Beilstein_J_Org_Chem-21-839-s001.pdf]

BEILSTEIN JOURNAL OF ORGANIC CHEMISTRY

Supporting Information

for

Chitosan-supported CuI-catalyzed cascade reaction of 2-halobenzoic acids and amidines for the synthesis of quinazolinones

Xuhong Zhao, Weishuang Li, Mengli Yang, Bojie Li, Yaoyao Zhang, Lizhen Huang and Lei Zhu

Beilstein J. Org. Chem. **2025,** *21,* 839–844. doi:10.3762/bjoc.21.67

Full experimental details, characterization data and copies of NMR spectra of all products

License and Terms: This is a supporting information file under the terms of the Creative Commons Attribution License (https://creativecommons.org/licenses/by/4.0). Please note that the reuse, redistribution and reproduction in particular requires that the author(s) and source are credited and that individual graphics may be subject to special legal provisions.
The license is subject to the *Beilstein Journal of Organic Chemistry* terms and conditions: (https://www.beilstein-journals.org/bjoc/terms)

Contents

General information and materials S1

General procedure for the preparation of chitosan-supported on CuI S1

General procedure for the preparation of the quinazolinones S3

References S7

^{1}H NMR and ^{13}C NMR spectra S9

General information and materials

Unless otherwise stated, all experiments were carried out open in the air. Reactions were monitored by thin-layer chromatography (TLC). TLC was performed using Huanghai 8 ± 0.2 μm precoated glass plates (0.25 mm) and visualized by UV fluorescence quenching, $KMnO_4$, or phosphomolybdic acid staining. Huanghai silica gel (200–300 mess) was used for chromatography. ^{1}H NMR spectra were recorded at room temperature on a Bruker Advance III 400 MHz spectrometer, and were reported relative to residual $CDCl_3$ (δ 7.26 ppm). ^{13}C NMR spectra were recorded on a Bruker Advance III 400 MHz spectrometer (100 MHz) and were reported relative to $CDCl_3$ (δ 77.16 ppm). Data for ^{1}H NMR and ^{13}C NMR were reported as chemical shift (δ ppm) (multiplicity, coupling constant (Hz), integration) using standard abbreviations for multiplicities: s = singlet, d = doublet, t = triplet, q = quartet, quint = quintet, m = multiplet, and brs = broad signal. Unless otherwise noted, all reagents were purchased commercially and used without further purification. Petroleum ether (PE) (60–90 °C) and ethyl acetate (EA) were used as eluent for silica gel chromatography.

General procedure for the preparation of chitosan-supported on CuI

General procedure: Preparation method according to known literature, [1]to a 20 mL flask equipped with a magnetic stirring bar were added CuI (500.2 mg), chitosan (499.8 mg) and H_2O (10.0 mL), the whole system was stirred at room temperature for 3 h. After completion of the reaction, filtered and washed with water (50 mL). Then the filter residue was dried at 50 °C to obtain the chitosan-supported on CuI (CS@CuI) and the content of copper in the catalyst was 14.6% (2.3 mmol/g) measured by Inductively Coupled Plasma (ICP) atomic emission spectrometry. At the same time, the catalytic material was characterized by XRD, the results show that the CS@CuI diffraction peak corresponds to CuI standard card (JCPDS, 06-0246), indicating that the copper ions on the catalyst

are mainly in the form of CuI (Scheme S1).

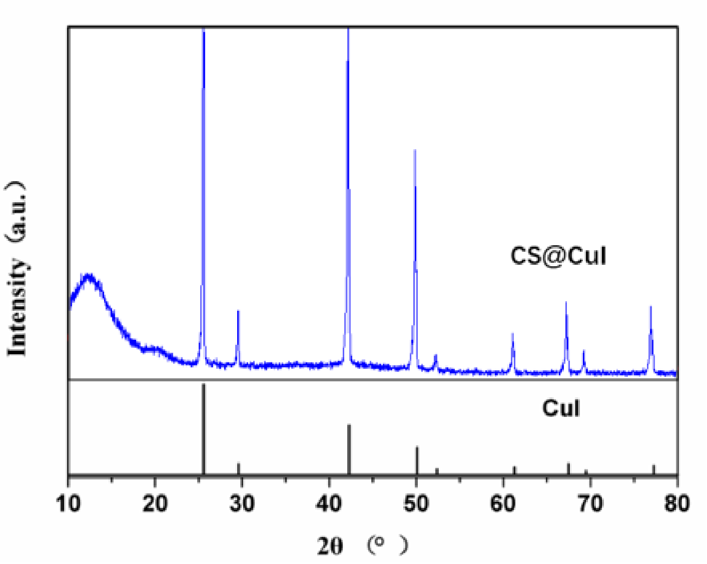

Scheme S1 XRD spectra of CS@CuI

The morphology of the resulting products was characterized using scanning electron microscopy (SEM) and EDS elemental mapping. The SEM analysis (Scheme S2a) and EDS elemental mapping (Scheme S2b) both confirmed a uniform distribution of CuI throughout the chitosan matrix. Specifically, CuI accounts for 92.9 wt % of the composition (Scheme S2c).

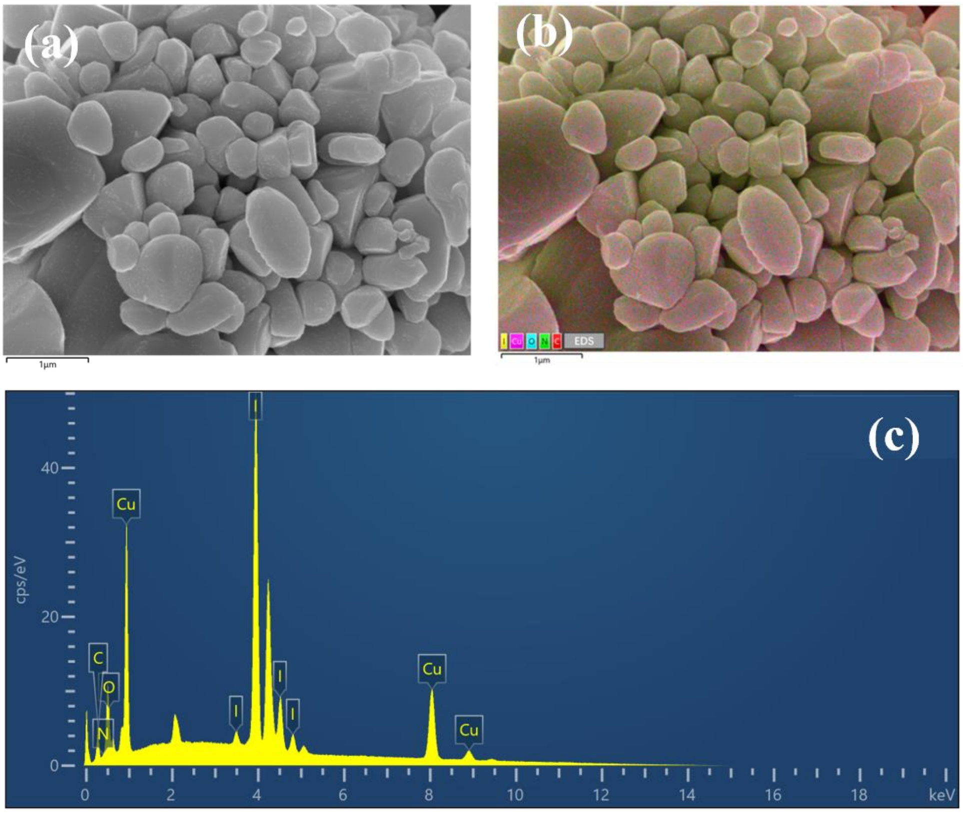

Total number of distribution maps				
Element	Line Type	Wt%	Wt% Sigma	At%
C	K-line system	6.31	0.10	34.35
N	K-line system	0.00	0.07	0.00
O	K-line system	0.73	0.04	2.97
Cu	L-line system	28.70	0.12	29.54
I	L-line system	64.20	0.13	33.13
Total		100.00		100.00

Scheme S2 SEM and EDS spectra of CS@CuI

General procedure for the preparation of the quinazolinones

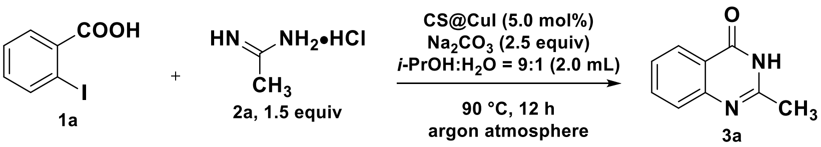

General procedure: Under argon atmosphere, to a 3.0 mL reaction tube equipped with a magnetic stirring bar were added **1a** (124.1 mg, 0.5 mmol), amidines hydrochloride (70.7 mg, 0.75 mmol, 1.5 equiv), CS@CuI (10.8 mg,

5.0 mol %), Na_2CO_3 (132.6 mg, 1.25 mmol) and 2.0 mL of mixed solvents (iPrOH/H_2O = 9:1). The whole reaction was stirred at 90 °C for 12 h. After completion the reaction, it was cooled to room temperature, quenched with H_2O and filtered through celite. The whole aqueous solution was extracted with EA (10 mL × 3), separated and combined the organic phase, then washed with brine, dried over anhydrous Na_2SO_4, filtered and the organic solvents were removed under vacuum and the desired product **3a** (96% yield) was obtained as a white solid after purification by silica gel chromatography (PE/EA = 5:1). ^{1}H NMR (400 MHz, DMSO-d_6) δ 12.20 (brs, 1H), 8.07 (d, J = 6.3 Hz, 1H), 7.76 (t, J = 7.7 Hz, 1H), 7.56 (d, J = 8.1 Hz, 1H),7.45 (t, J = 7.2 Hz, 1H), 2.34 (s, 3H); ^{13}C NMR (100 MHz, DMSO-d_6) δ 162.2, 154.7, 149.4, 134.7, 127.0, 126.3, 126.1, 121.1, 21.9. [2]

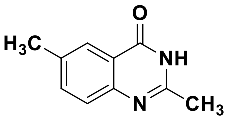

3b

2,6-Dimethylquinazolin-4(3*H*)-one (**3b**): ^{1}H NMR (400 MHz, DMSO-d_6) δ 12.10 (brs, 1H), 7.86 (s, 1H), 7.58 (d, J = 8.2 Hz, 1H), 7.47 (d, J = 8.2 Hz, 1H), 2.42 (s, 3H), 2.33 (s, 3H); ^{13}C NMR (100 MHz, DMSO-d_6) δ 161.7, 153.3, 146.9, 135.5, 135.4, 126.4, 125.0, 120.4, 21.4, 20.7. [3]

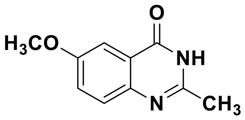

3c

6-Methoxy-2-methylquinazolin-4(3*H*)-one (**3c**): ^{1}H NMR (400 MHz, DMSO-d_6) δ 12.18 (brs, 1H), 7.52 (d, J = 8.9 Hz, 1H), 7.45 (d, J = 3.0 Hz, 1H), 7.37 (dd, J = 8.9, 3.0 Hz, 1H), 3.84 (s, 3H), 2.31 (s, 3H); ^{13}C NMR (100 MHz, DMSO-d_6) δ 161.5, 157.1, 151.8, 143.5, 128.3, 123.7, 121.3, 105.7, 55.5, 21.2. [4]

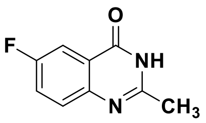

3d

6-Fluoro-2-methylquinazolin-4(3*H*)-one (**3d**): ^{1}H NMR (400 MHz, DMSO-d_6) δ 12.38 (brs, 1H), 7.80 – 7.74 (m, 1H), 7.73 – 7.66 (m, 2H), 2.38 (s, 3H); ^{13}C NMR (100 MHz, DMSO-d_6) δ 161.2 (J_{C-F} = 3.9 Hz), 159.6 (J_{C-F} = 242.7 Hz), 153.8 (J_{C-F} = 2.0 Hz), 145.9 (J_{C-F} = 1.8 Hz), 129.4 (J_{C-F} = 8.3 Hz), 122.7 (J_{C-F} = 23.7 Hz), 121.8 (J_{C-F} = 2.8 Hz), 110.3 (J_{C-F} = 23.1 Hz), 21.4. [5]

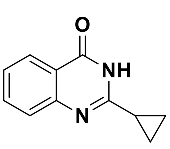

3e

2-Cyclopropylquinazolin-4(3*H*)-one (**3e**): 1H NMR (400 MHz, DMSO-d_6) δ 12.46 (brs, 1H), 8.04 (d, *J* = 6.4 Hz, 1H), 7.72 (t, *J* = 8.5 Hz, 1H), 7.48 (d, *J* = 7.7 Hz, 1H), 7.40 (t, *J* = 8.1 Hz, 1H), 2.01 – 1.89 (m, 1H), 1.13 – 1.07 (m, 2H), 1.06 – 0.98 (m, 2H); ^{13}C NMR (100 MHz, DMSO-d_6) δ 161.6, 159.0, 149.1, 134.3, 126.5, 125.8, 125.3, 120.6, 13.5, 9.5. [6]

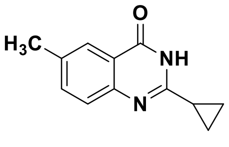

3f

2-Cyclopropyl-6-methylquinazolin-4(3*H*)-one (**3f**): ^{1}H NMR (400 MHz, DMSO-d_6) δ 12.35 (brs, 1H), 7.84 (s, 1H), 7.55 (d, *J* = 8.3 Hz, 1H), 7.38 (d, *J* = 8.3 Hz, 1H), 2.40 (s, 3H), 2.00 – 1.87 (m, 1H), 1.10 – 1.05 (m, 2H), 1.04 – 0.97 (m, 2H); ^{13}C NMR (100 MHz, DMSO-d_6) δ 162.0, 158.5, 147.6, 136.0, 135.3, 126.8, 125.6, 120.8, 21.2, 13.8, 9.8. [7]

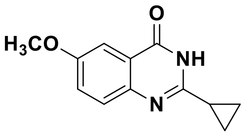

3g

2-Cyclopropyl-6-methoxyquinazolin-4(3*H*)-one (**3g**): ^{1}H NMR (400 MHz, DMSO-d_6) δ 12.40 (brs, 1H), 7.44 (s, 1H), 7.43 (d, *J* = 5.2 Hz, 1H), 7.33 (dd, *J* = 9.0, 3.0 Hz, 1H), 3.83 (s, 3H), 1.97 – 1.86 (m, 1H), 1.08 – 1.02 (m, 2H), 1.01 – 0.94 (m, 2H); ^{13}C NMR (100 MHz, DMSO-d_6) δ 161.9, 157.2, 157.0, 144.1, 128.6, 124.3, 121.7, 106.1, 55.9, 13.7, 9.6. [8]

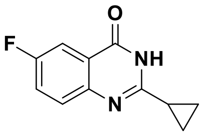

3h

2-Cyclopropyl-6-fluoroquinazolin-4(3*H*)-one (**3h**): ^{1}H NMR (400 MHz, DMSO-d_6) δ 12.6 (brs, 1H), 7.71 (dd, *J* = 8.7, 3.0 Hz, 1H), 7.65 – 7.57 (m, 1H), 7.56 – 7.51 (m, 1H), 2.01 – 1.89 (m, 1H), 1.11 – 1.05 (m, 2H), 1.04 – 0.98 (m, 2H); ^{13}C NMR (100 MHz, DMSO-d_6) δ 161.1, 159.2 ($J_{C\text{-}F}$ = 243.1 Hz), 158.0, 146.0, 129.2 ($J_{C\text{-}F}$ = 8.0 Hz), 122.7 ($J_{C\text{-}F}$ = 23.8 Hz), 121.7 ($J_{C\text{-}F}$ = 8.0 Hz), 110.3 ($J_{C\text{-}F}$ = 22.9 Hz), 13.4, 9.6. [9]

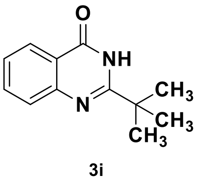

3i

2-(*tert*-Butyl)quinazolin-4(3*H*)-one (**3i**): ^{1}H NMR (400 MHz, DMSO-d_6) δ 11.9 (brs, 1H), 8.09 (d, *J* = 7.9 Hz, 1H), 7.78 (t, *J* = 8.5 Hz, 1H), 7.61 (d, *J* = 7.7 Hz, 1H), 7.47 (t, *J* = 8.1 Hz, 1H),

1.35 (s, 9H); ^{13}C NMR (100 MHz, DMSO-d_6) δ 163.1, 162.8, 148.8, 134.8, 127.8, 126.7, 126.1, 121.1, 37.7, 28.3. [7]

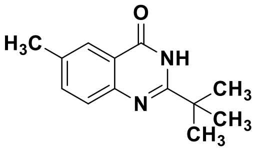

3j

2-(*tert*-Butyl)-6-methylquinazolin-4(3*H*)-one (**3j**): ^{1}H NMR (400 MHz, DMSO-d_6) δ 11.83 (brs, 1H), 7.89 (s, 1H), 7.60 (d, J = 8.4 Hz, 1H), 7.52 (d, J = 8.2 Hz, 1H), 2.43 (s, 3H), 1.34 (s, 9H); ^{13}C NMR (100 MHz, DMSO-d_6) δ 162.7, 162.2, 146.8, 136.2, 136.0, 127.6, 125.4, 120.8, 37.6, 28.3, 21.2. [10]

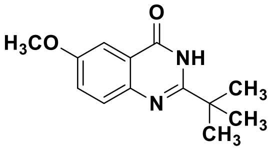

3k

2-(*tert*-Butyl)-6-methoxyquinazolin-4(3*H*)-one (**3k**): ^{1}H NMR (400 MHz, DMSO-d_6) δ 11.86 (s, 1H), 7.56 (d, J = 8.8 Hz, 1H), 7.48 (d, J = 3.0 Hz, 1H), 7.37 (dd, J = 8.9, 3.0 Hz, 1H), 3.86 (s, 3H), 1.34 (s, 9H); ^{13}C NMR (100 MHz, DMSO-d_6) δ 162.6, 160.8, 157.8, 143.2, 129.4, 124.2, 121.8, 106.0, 56.0, 37.5, 28.3. [10]

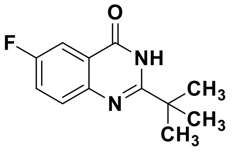

3l

2-(*tert*-Butyl)-6-fluoroquinazolin-4(3*H*)-one (**3l**): ^{1}H NMR (400 MHz, DMSO-d_6) δ 12.09 (brs, 1H), 7.80 (dd, J = 8.5, 2.8 Hz, 1H), 7.76 – 7.66 (m, 2H), 1.39 (s, 9H); ^{13}C NMR (100 MHz, DMSO-d_6) δ 162.6 ($J_{C\text{-}F}$ = 2.2 Hz), 162.2 ($J_{C\text{-}F}$ = 3.6 Hz), 160.2 ($J_{C\text{-}F}$ = 243.1 Hz), 145.6 ($J_{C\text{-}F}$ = 1.8 Hz), 130.6 ($J_{C\text{-}F}$ = 8.3 Hz), 123.2 ($J_{C\text{-}F}$ = 23.9 Hz), 122.3 ($J_{C\text{-}F}$ = 4.8 Hz), 110.7 ($J_{C\text{-}F}$ = 23.0 Hz), 37.7, 28.2. [10]

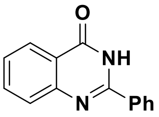

3m

2-Phenylquinazolin-4(3*H*)-one (**3m**): ^{1}H NMR (400 MHz, DMSO-d_6) δ 12.43 (brs, 1H), 8.27 – 8.09 (m, 3H), 7.85 (t, J = 7.6 Hz, 1H), 7.75 (d, J = 8.0 Hz, 1H), 7.65 – 7.46 (m, 4H); ^{13}C NMR (100 MHz, DMSO-d_6) δ 162.3, 152.4, 148.7, 134.6, 132.7, 131.4, 128.6, 127.8, 127.4, 126.6, 125.9, 121.0. [11]

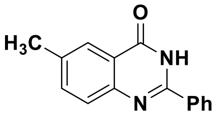

3n

6-Methyl-2-phenylquinazolin-4(3*H*)-one (**3n**): ^{1}H NMR (400 MHz, DMSO-d_6) δ 12.45 (brs, 1H), 8.22 – 8.11 (m, 2H), 7.95 (s, 1H), 7.68 – 7.62 (m, 2H), 7.61 – 7.49 (m, 3H), 2.46 (s, 3H); ^{13}C NMR (100 MHz, DMSO-d_6) δ 162.2, 151.5, 146.7, 136.3, 135.9, 132.8,

131.2, 128.6, 127.6, 127.4, 125.3, 120.7, 20.9. [3]

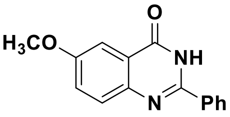

3o

6-Methoxy-2-phenylquinazolin-4(3*H*)-one (**3o**): ^{1}H NMR (400 MHz, DMSO-d_6) δ 12.50 (brs, 1H), 8.22 – 8.11 (m, 2H), 7.70 (d, *J* = 8.9 Hz, 1H), 7.61 – 7.49 (m, 4H), 7.44 (dd, *J* = 8.9, 3.0 Hz, 1H), 3.89 (s, 3H); ^{13}C NMR (100 MHz, DMSO-d_6) δ 162.1, 157.7, 150.2, 143.2, 132.8, 131.1, 129.2, 128.6, 127.5, 124.1, 121.8, 105.9, 55.7. [12]

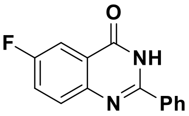

3p

6-Fluoro-2-phenylquinazolin-4(3*H*)-one (**3p**): ^{1}H NMR (400 MHz, DMSO-d_6) δ 12.67 (brs, 1H), 8.16 (d, *J* = 6.7 Hz, 2H), 7.88 – 7.78 (m, 2H), 7.76 – 7.68 (m, 1H), 7.63 – 7.51 (m, 3H); ^{13}C NMR (100 MHz, DMSO-d_6) δ 161.8, 160.0 ($J_{C\text{-}F}$ = 244.0 Hz), 151.9, 145.6, 132.6, 131.4, 130.3 ($J_{C\text{-}F}$ = 7.9 Hz), 128.6, 127.8, 123.1 ($J_{C\text{-}F}$ = 23.9 Hz), 122.2 ($J_{C\text{-}F}$ = 8.5 Hz), 110.5 ($J_{C\text{-}F}$ = 23.1 Hz). [5]

References

[1] Baig, R. B. N.; Varma, R. S. *Green Chem*. **2013**, *15*, 1839.

[2] Mondal, P. P.; Pal, A.; Prakash, A. K.; Sahoo, B. *Chem. Commun*. **2022**, *58*, 13202.

[3] Wang, X.; Lerchen, A.; Glorius, F. *Org. Lett*. **2016**, *18*, 2090.

[4] Pitta, E.; Balabon, O.; Rogacki, M. K.; Gómez, J.; Cunningham, F.; Joosens, J.; Augustyns, K.; Van der Veken, P.; Bates, R. *Eur. J. Med. Chem*. **2017**, *125*, 890.

[5] Xu, Y.; Xie, Q.; Li, W.; Sun, H.; Wang, Y.; Shao, L. *Tetrahedron* **2015**, *71*, 4853.

[6] Jia, F.-C.; Zhou, Z.-W.; Xu, C.; Wu, Y.-D.; Wu, A.-X. *Org. Lett.* **2016**, *18*, 2942.

[7] Li, Z.; Dong, J.; Chen, X.; Li, Q.; Zhou, Y.; Yin, S.-F. *J. Org. Chem*. **2015**, *80*, 9392.

[8] Peddibhotla, S.; Hedrick, M. P.; Hershberger, P.; Maloney, P.R.; Li, Y.; Milewski, M.; Gosalia, P.; Gray, W.; Mehta, A.; Sugarman,E.; Hood, B.; Suyama, E.; Nguyen, K.; Heynen-Genel, S.; Vasile, S.;Salaniwal, S.; Stonich, D.; Su, Y.; Mangravita-Novo, A.; Vicchiarelli,M.; Roth, G. P.; Smith, L. H.; Chung, T. D.; Hanson, G. R.; Thomas,J. B.; Caron, M. G.; Barak, L. S.; Pinkerton, A. B. *ACS Med. Chem. Lett*. **2013**, *4*, 846.

[9] Yu, L.; Wang, M.; Li, P.; Wang, L. *Appl. Organomet. Chem*. **2012**, *26*, 576.

[10] Zhu, L.; Han, B.; Wen, W.; Zhang, Y.; Li, B.; Xia, C.; Yan, Y.; Li, W.; Wang, L. CN112778218A.

[11] Lv, X.; Abrams, R.; Martin, R. *Angew. Chem., Int. Ed*. **2023**, *62*, No. e202217386.

[12] Xu, G.; Wang, L.; Li, M.; Tao, M.; Zhang, W. *Green Chem*. **2017**, *19*, 5818.

^{1}H NMR and ^{13}C NMR spectra

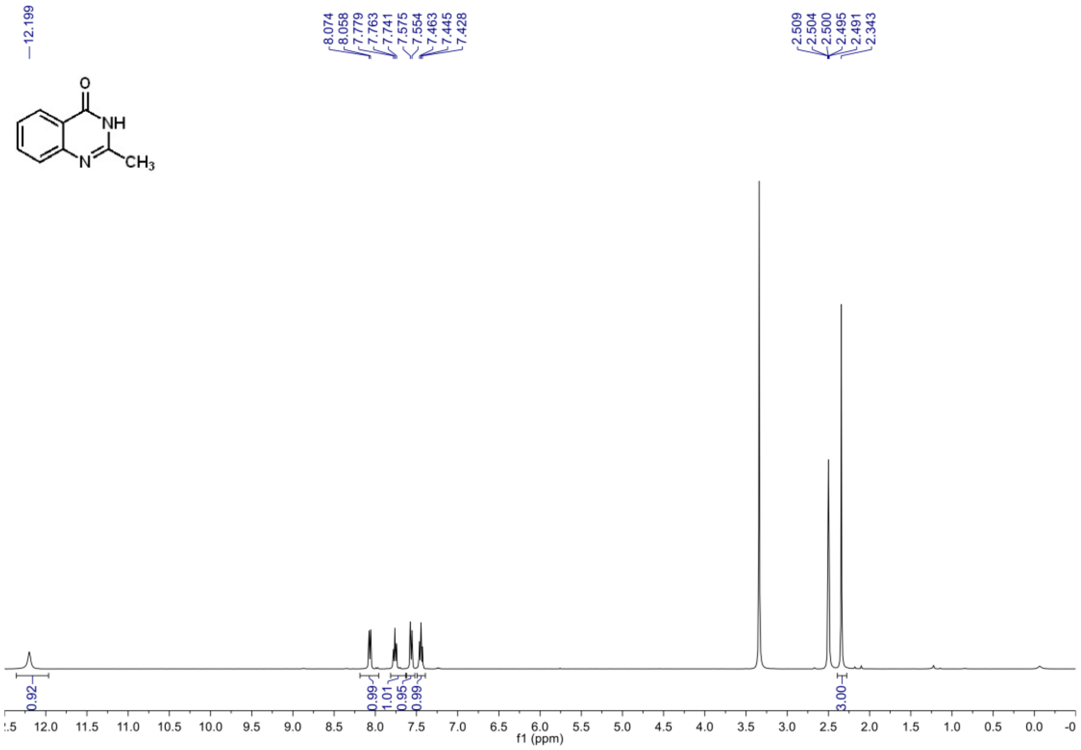

^{1}H NMR (400 MHz, DMSO-d_6) of compound **3a.**

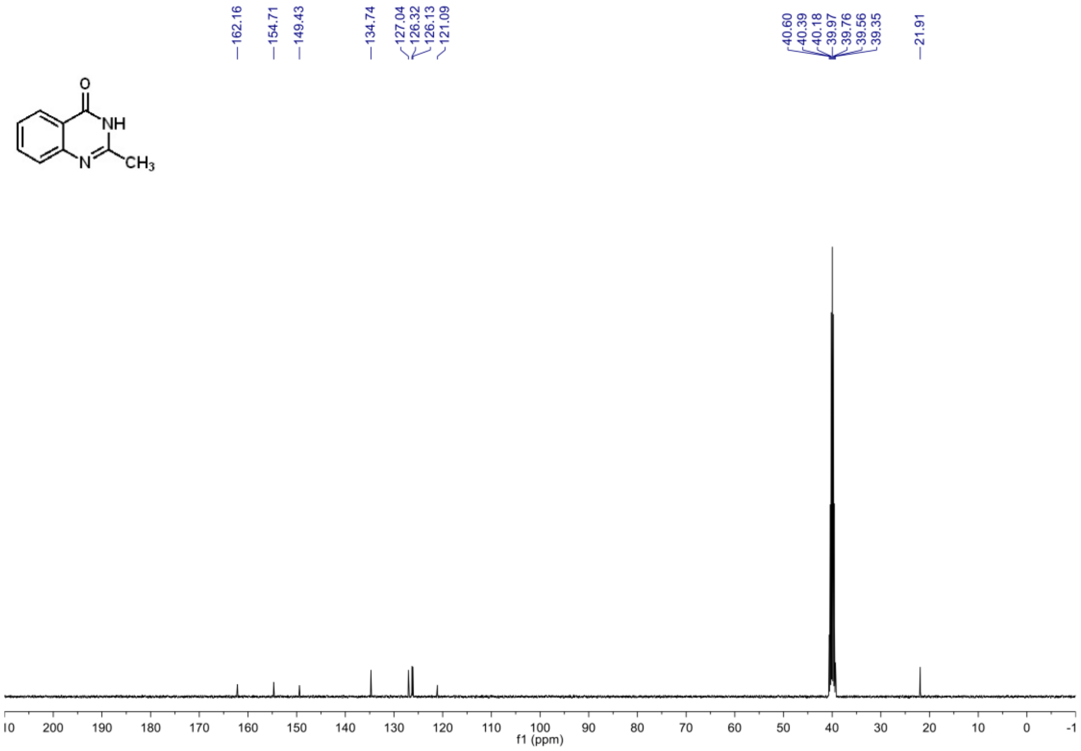

^{13}C NMR (100 MHz, DMSO-d_6) of compound **3a.**

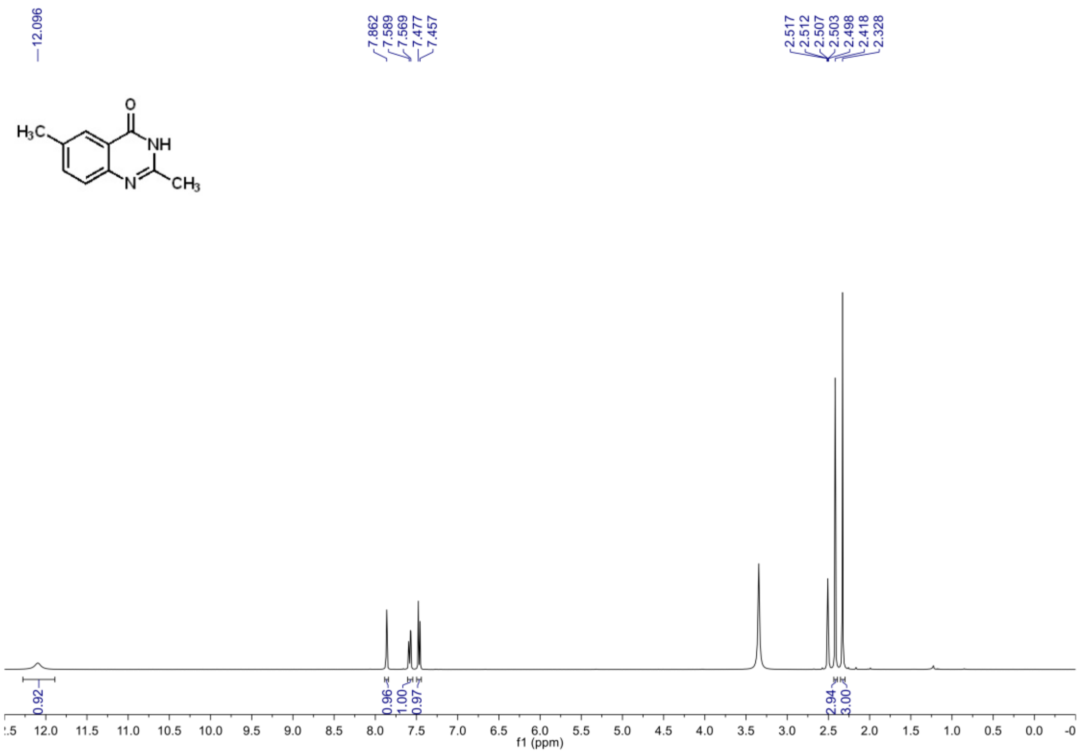

^{1}H NMR (400 MHz, DMSO-*d*$_6$) of compound **3b.**

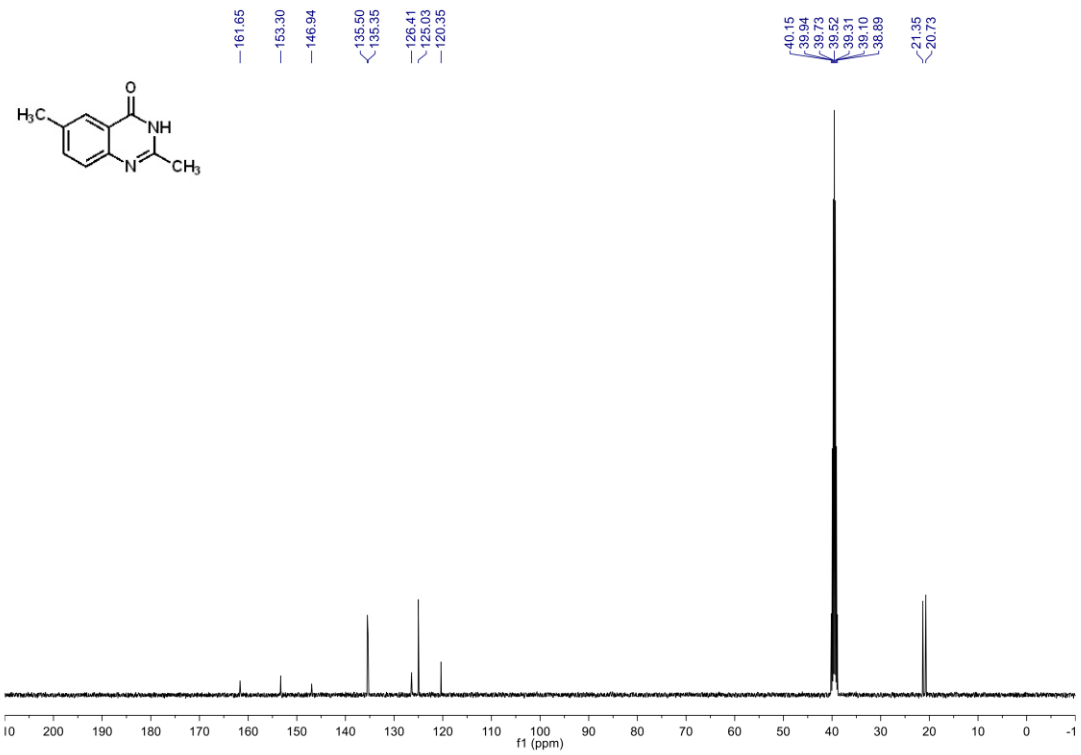

^{13}C NMR (100 MHz, DMSO-*d*$_6$) of compound **3b.**

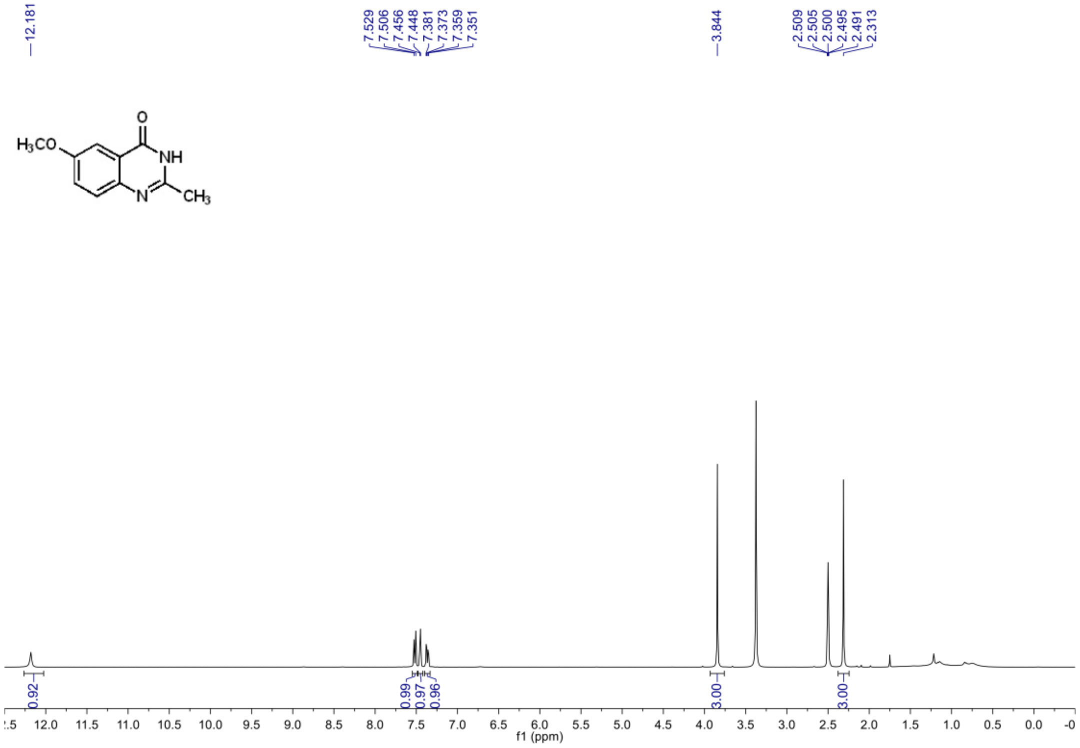

^{1}H NMR (400 MHz, DMSO-d_6) of compound **3c.**

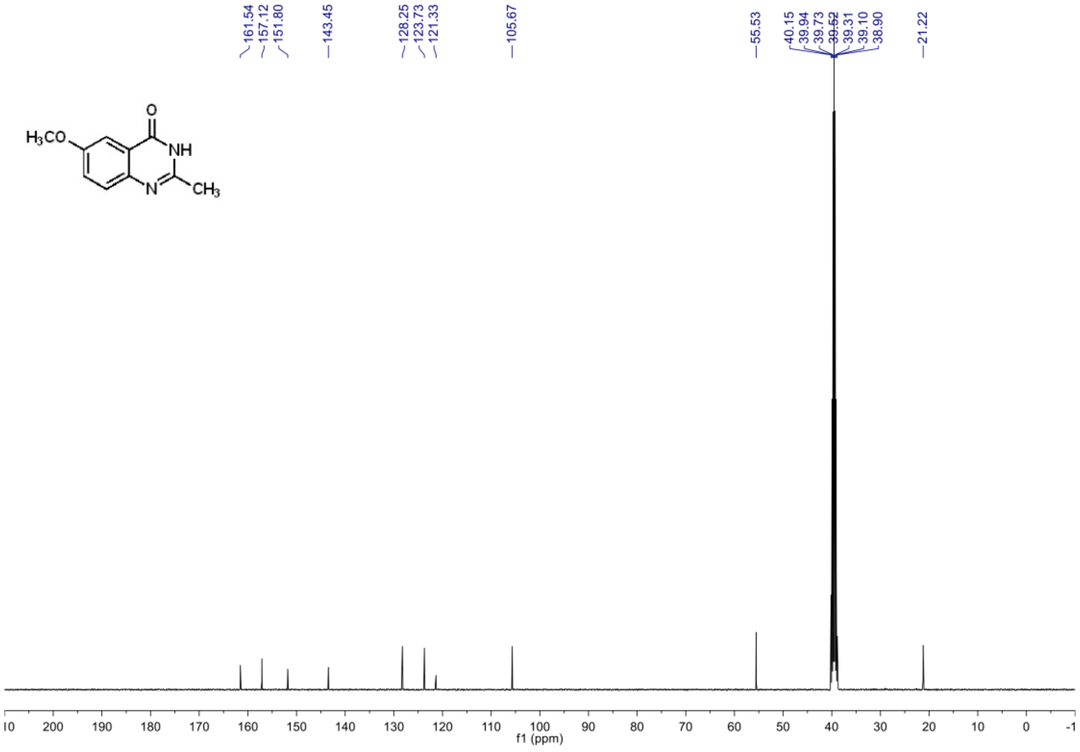

^{13}C NMR (100 MHz, DMSO-d_6) of compound **3c.**

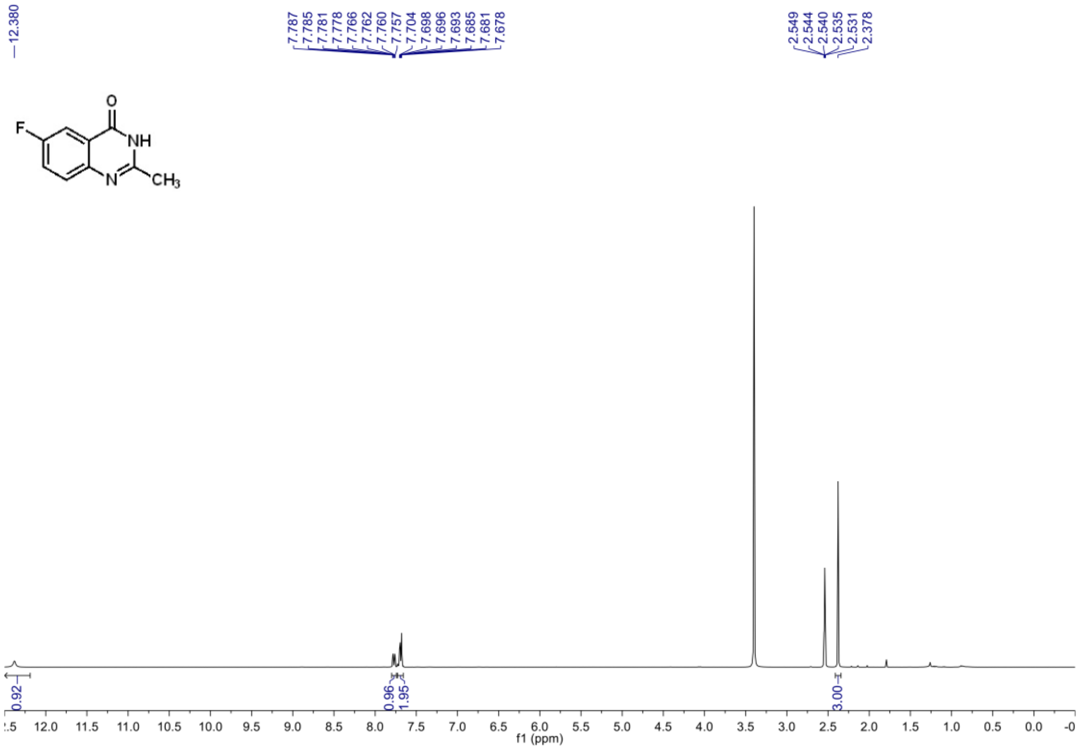

^{1}H NMR (400 MHz, DMSO-d_6) of compound **3d.**

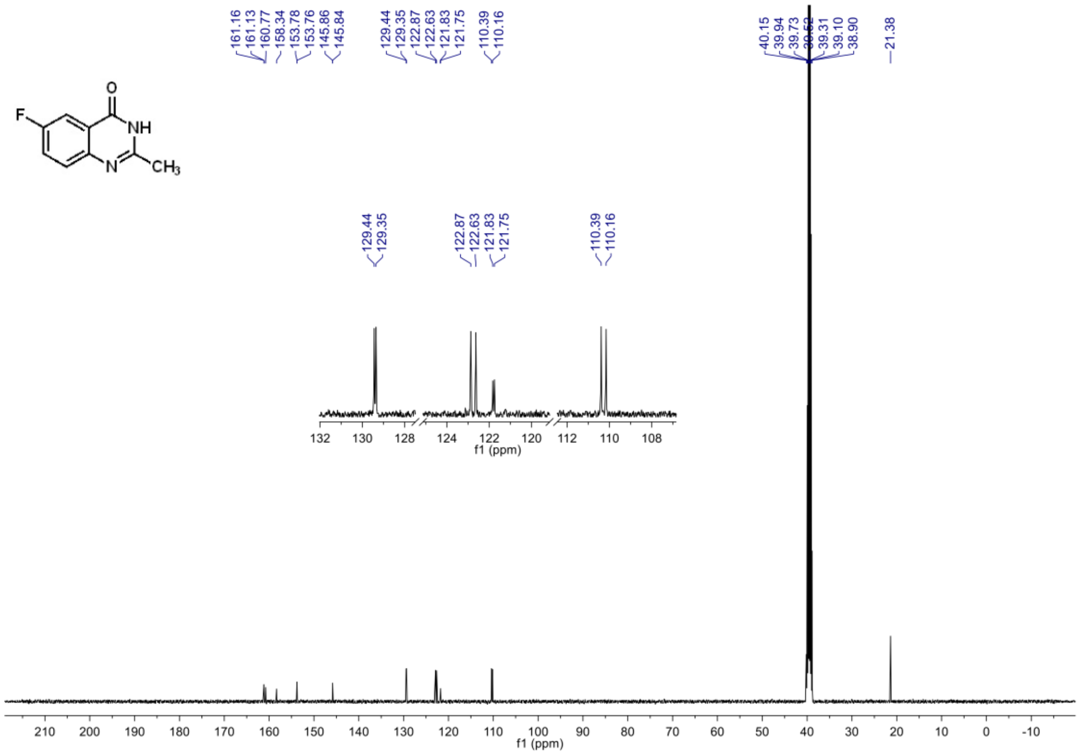

^{13}C NMR (100 MHz, DMSO-d_6) of compound **3d.**

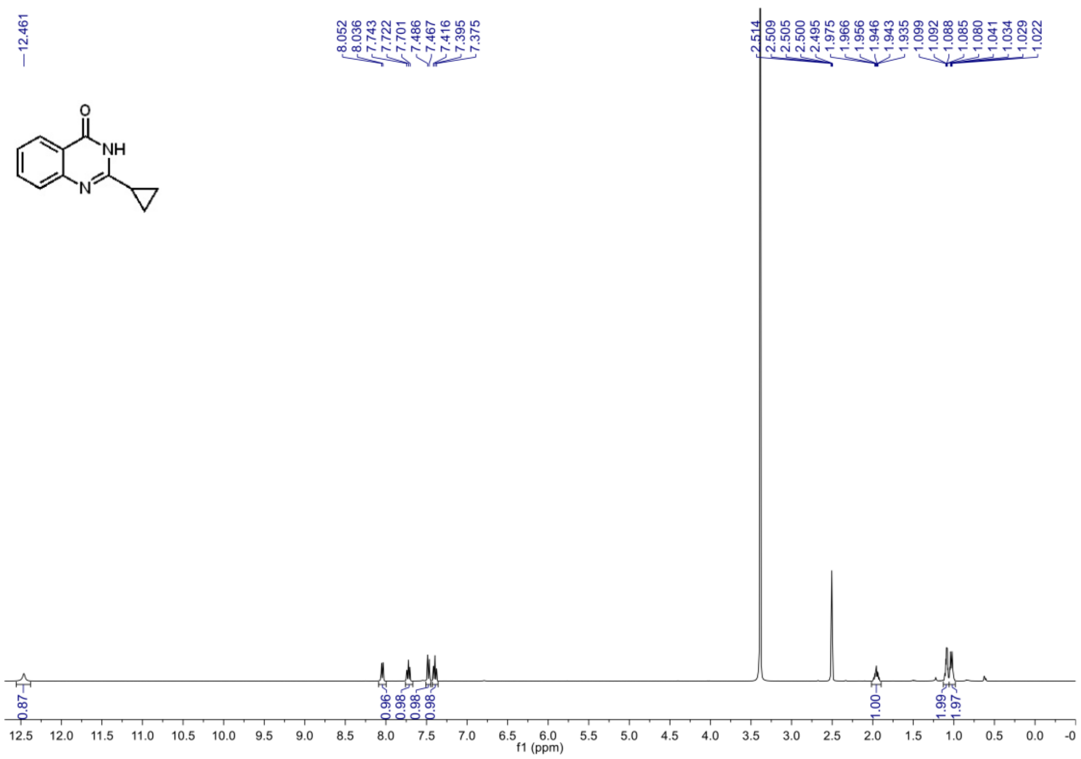

^{1}H NMR (400 MHz, DMSO-d_6) of compound **3e.**

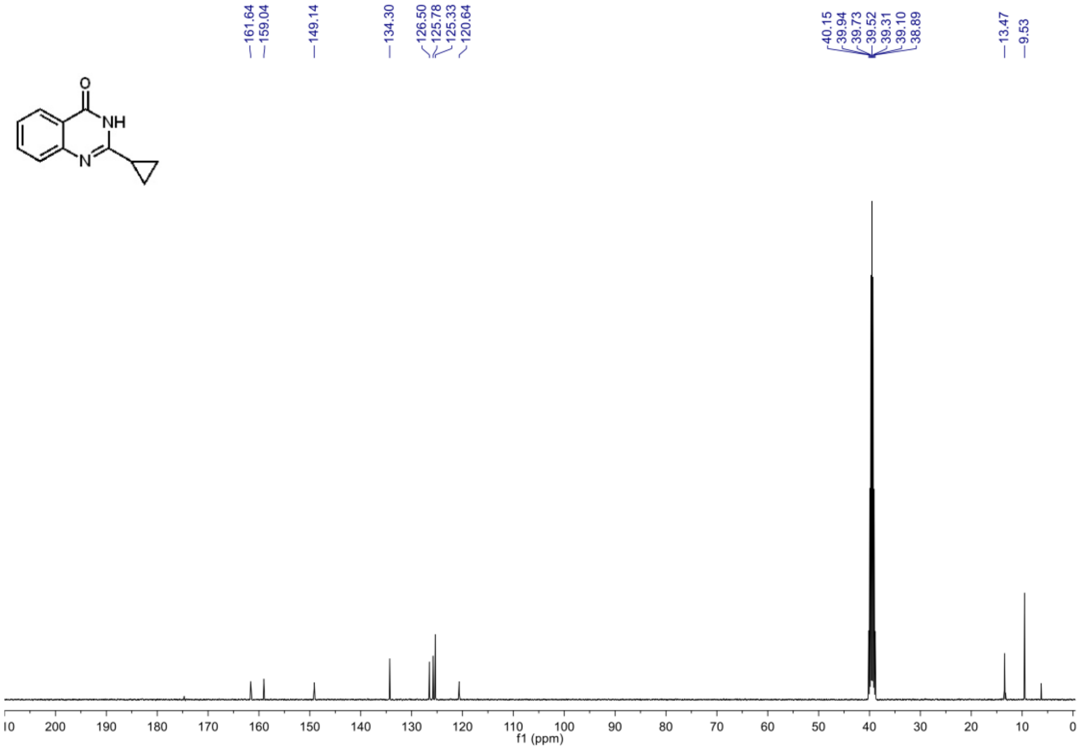

^{13}C NMR (100 MHz, DMSO-d_6) of compound **3e.**

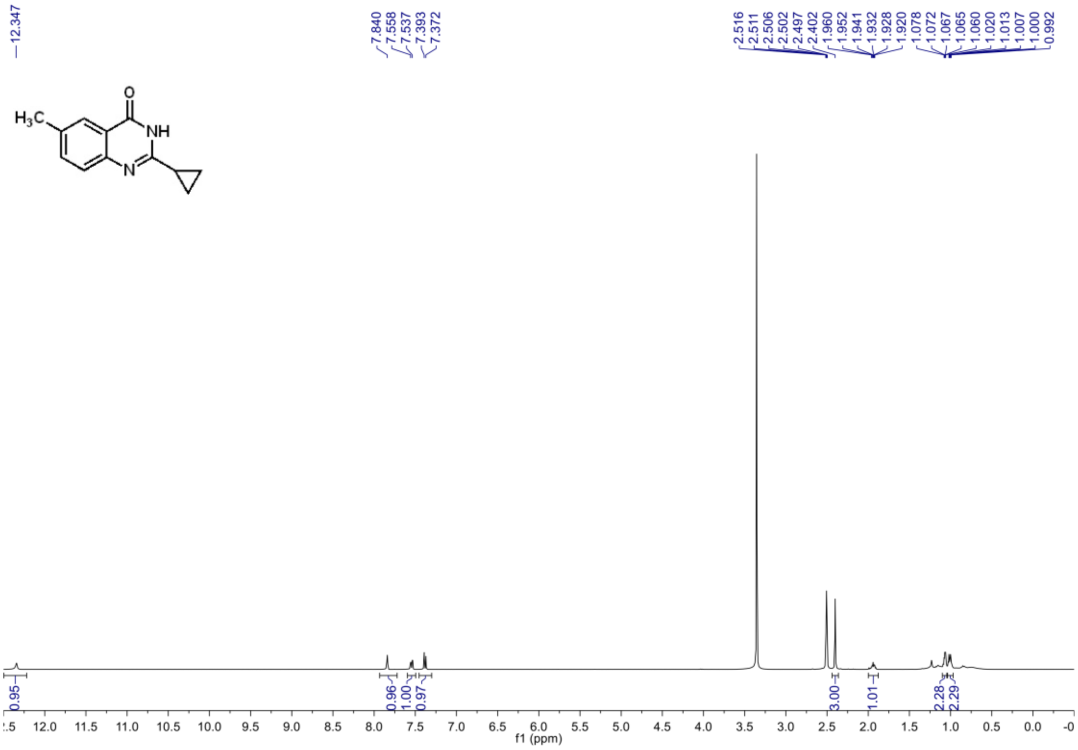

^{1}H NMR (400 MHz, DMSO-d_6) of compound **3f.**

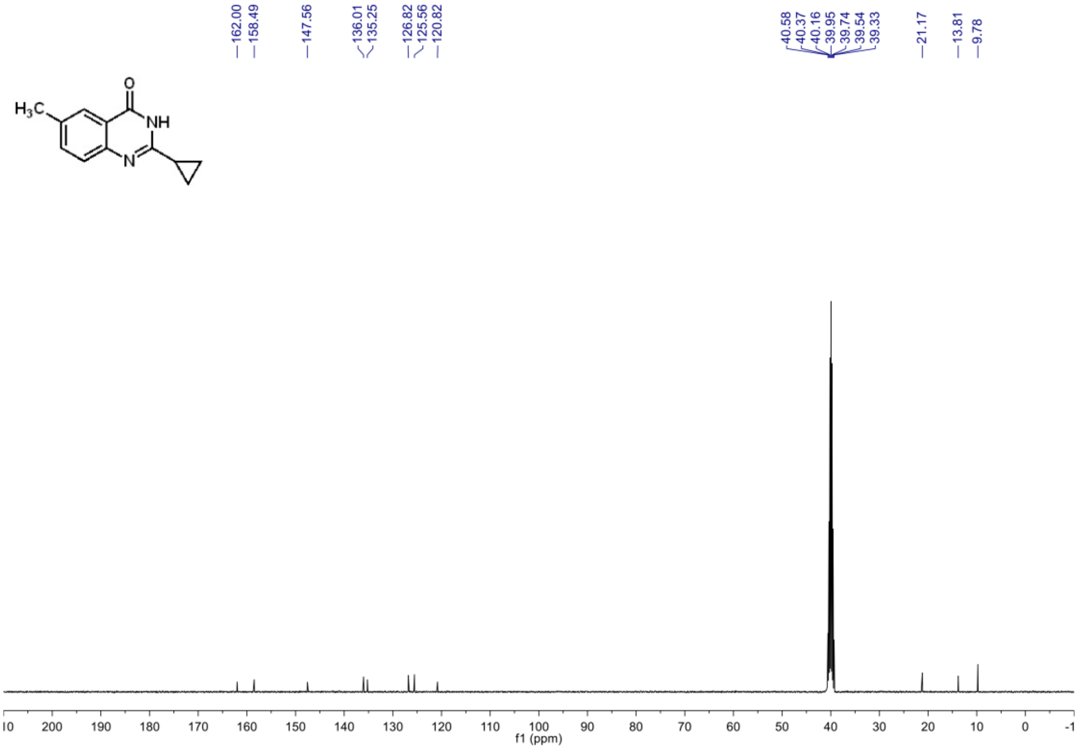

^{13}C NMR (100 MHz, DMSO-d_6) of compound **3f.**

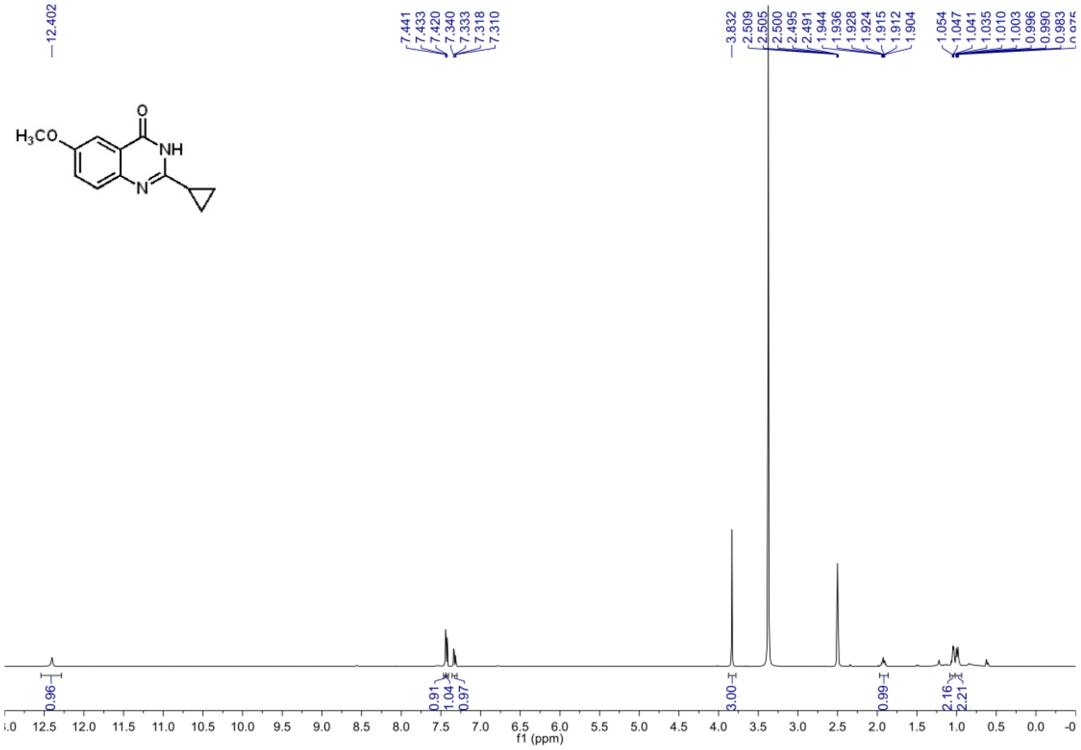

^{1}H NMR (400 MHz, DMSO-d_6) of compound **3g.**

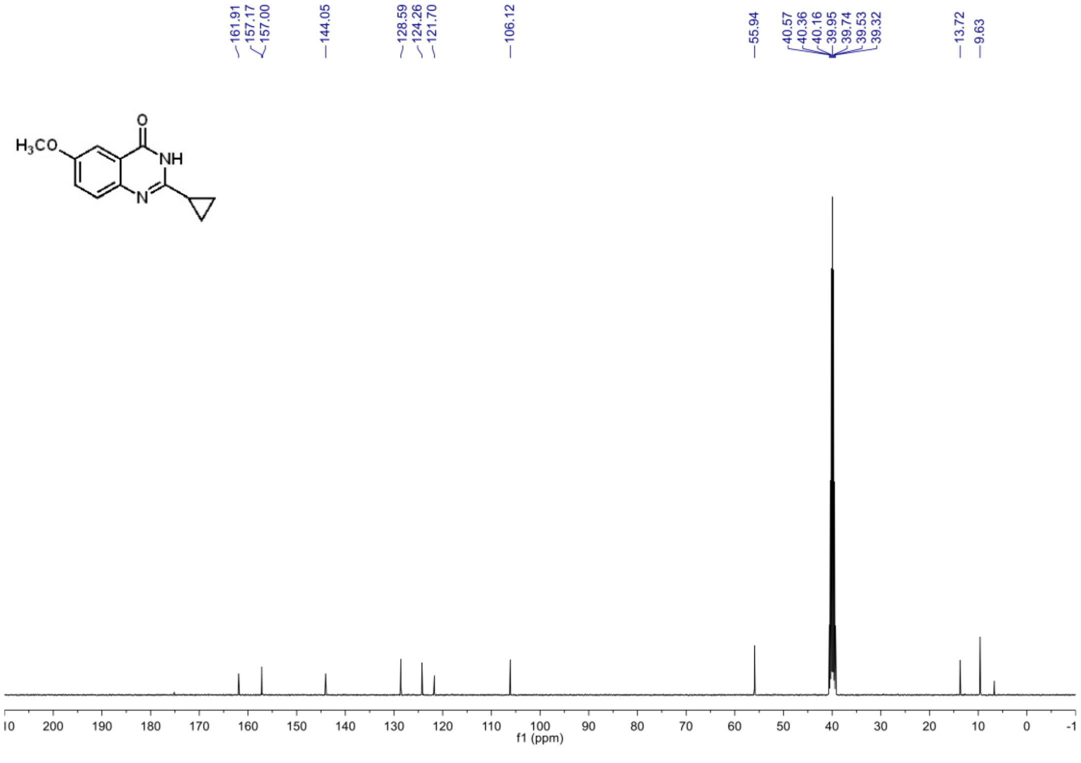

^{13}C NMR (100 MHz, DMSO-d_6) of compound **3g.**

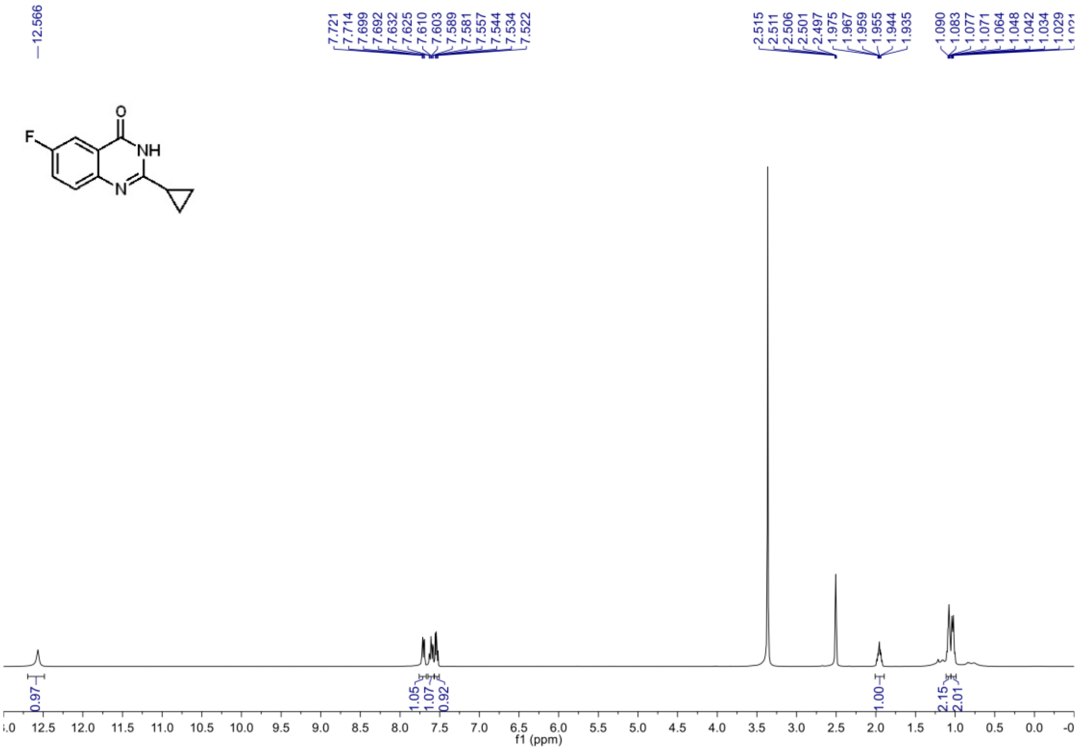

^{1}H NMR (400 MHz, DMSO-d_6) of compound **3h.**

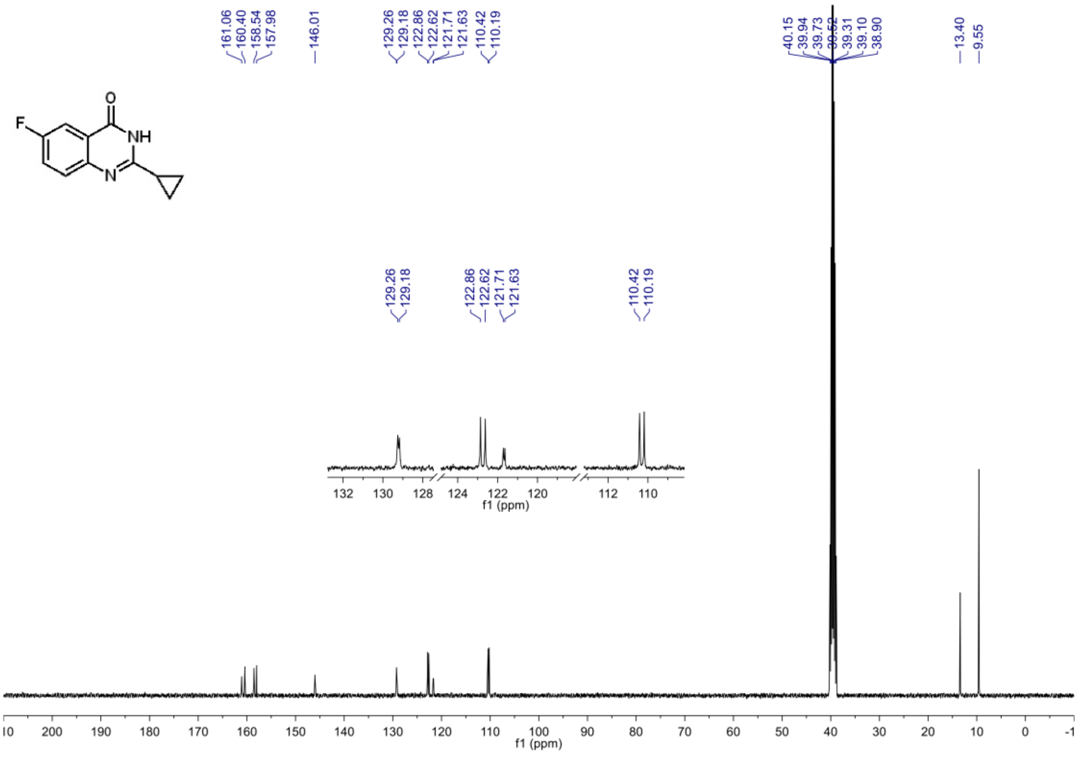

^{13}C NMR (100 MHz, DMSO-d_6) of compound **3h.**

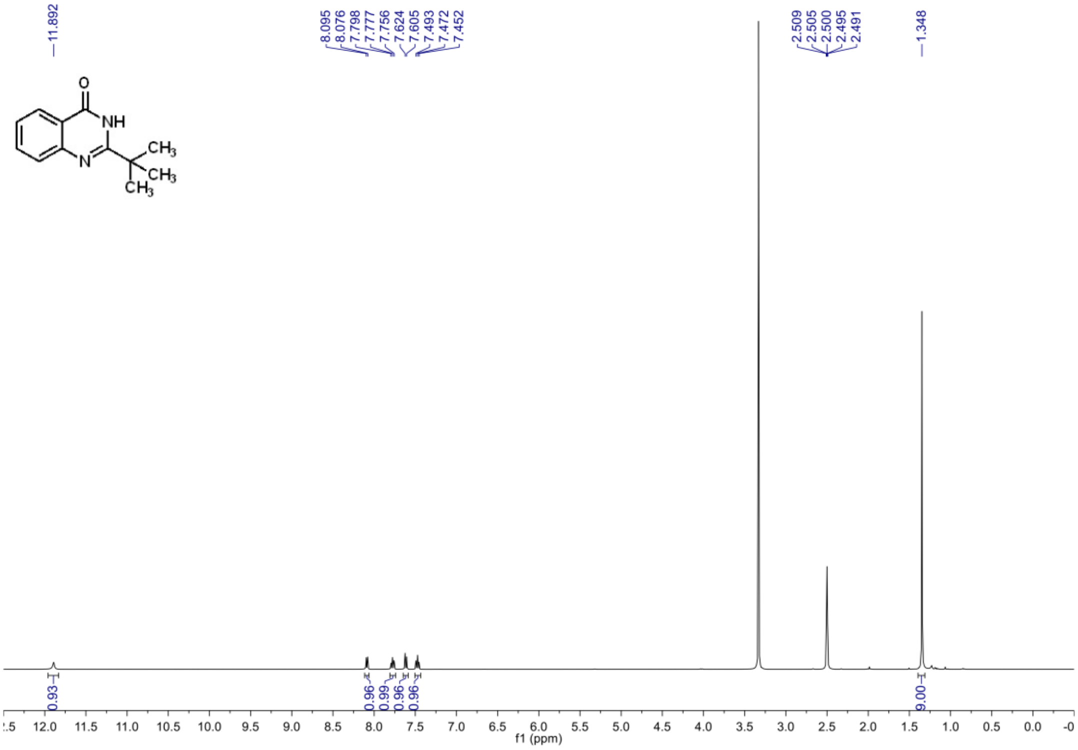

^{1}H NMR (400 MHz, DMSO-*d$_6$*) of compound **3i.**

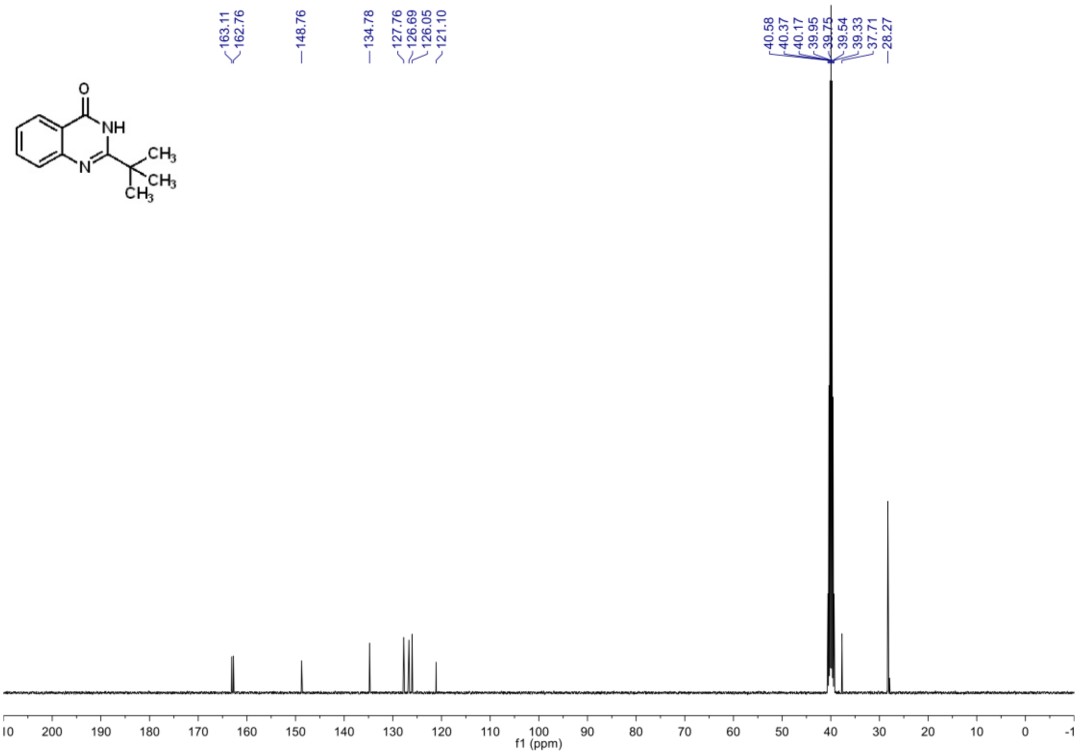

^{13}C NMR (100 MHz, DMSO-*d$_6$*) of compound **3i.**

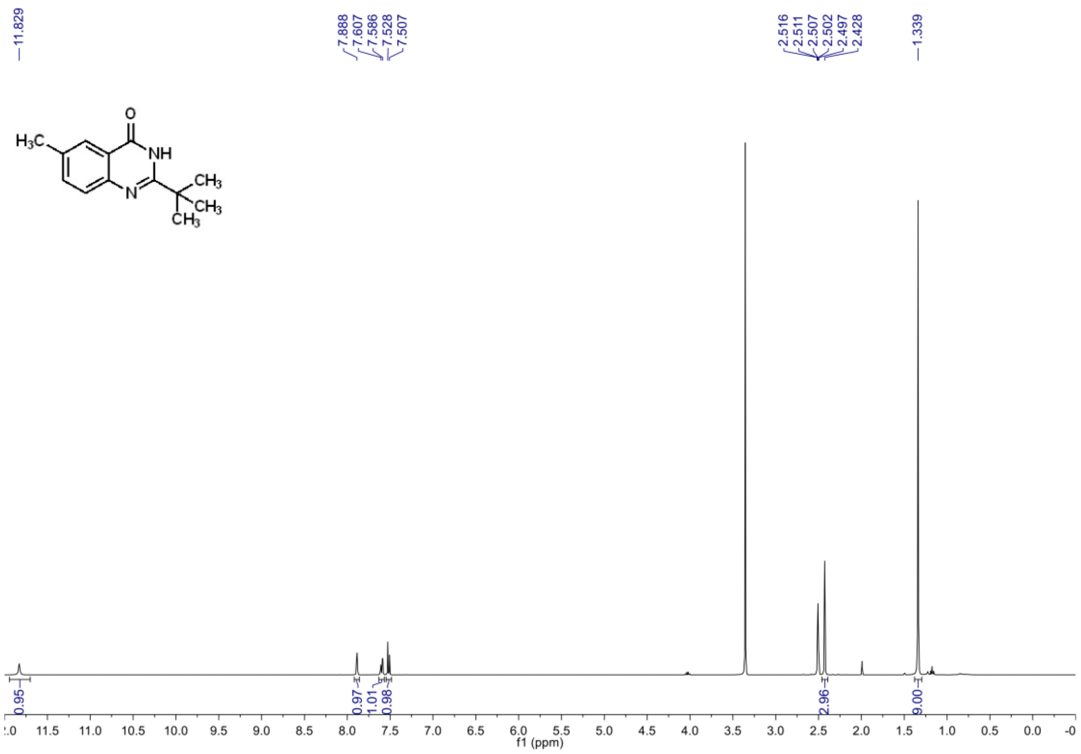

^{1}H NMR (400 MHz, DMSO-d_6) of compound **3j.**

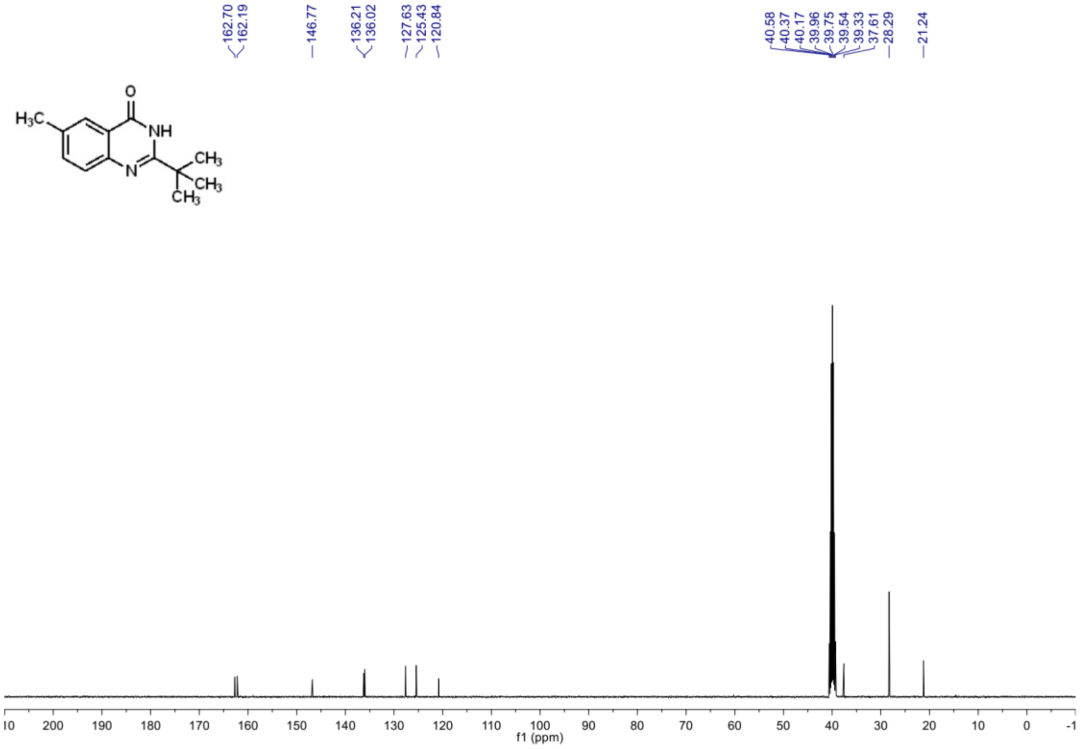

^{13}C NMR (100 MHz, DMSO-d_6) of compound **3j.**

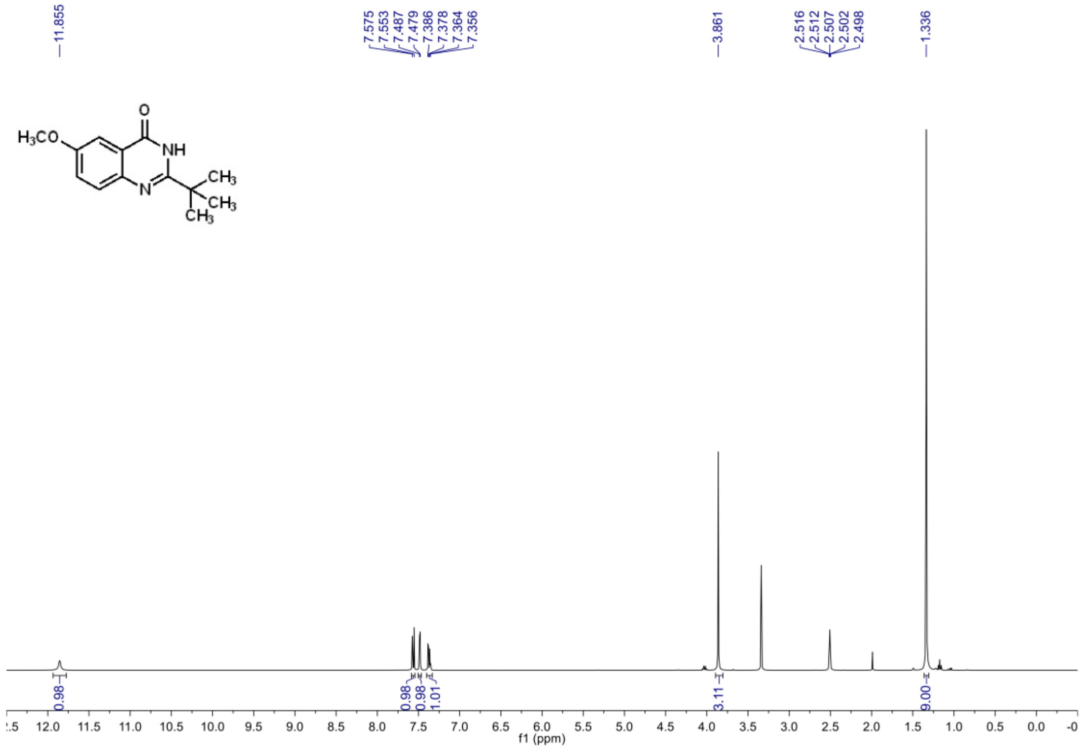

^{1}H NMR (400 MHz, DMSO-*d6*) of compound **3k.**

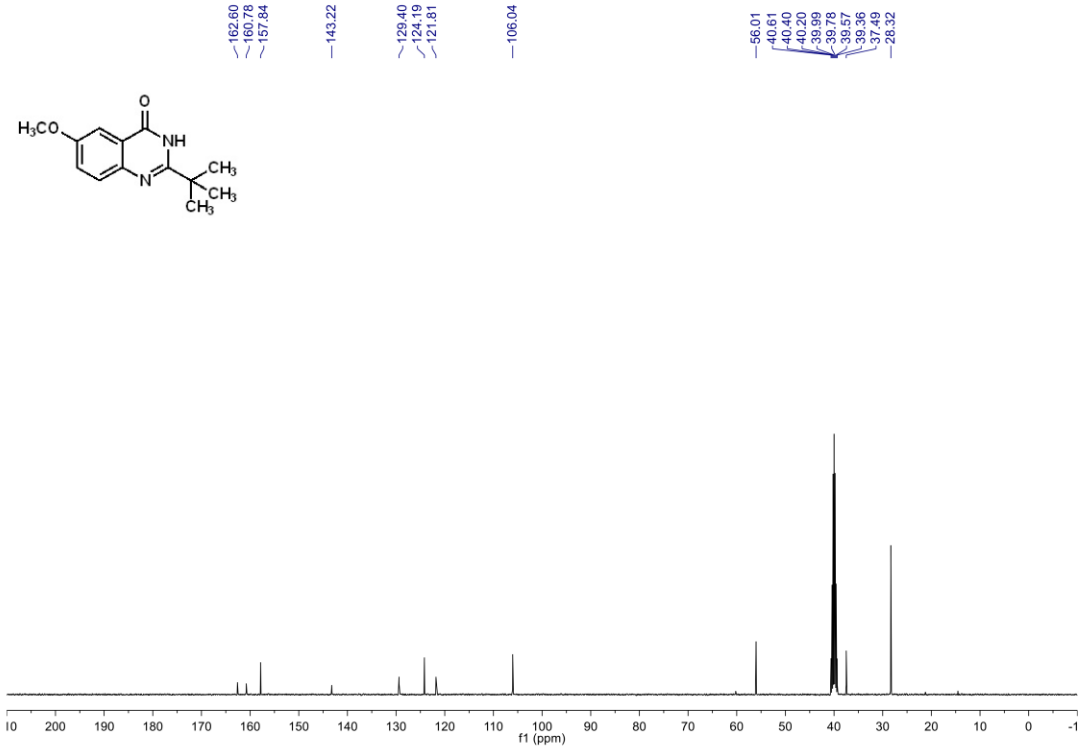

^{13}C NMR (100 MHz, DMSO-*d6*) of compound **3k.**

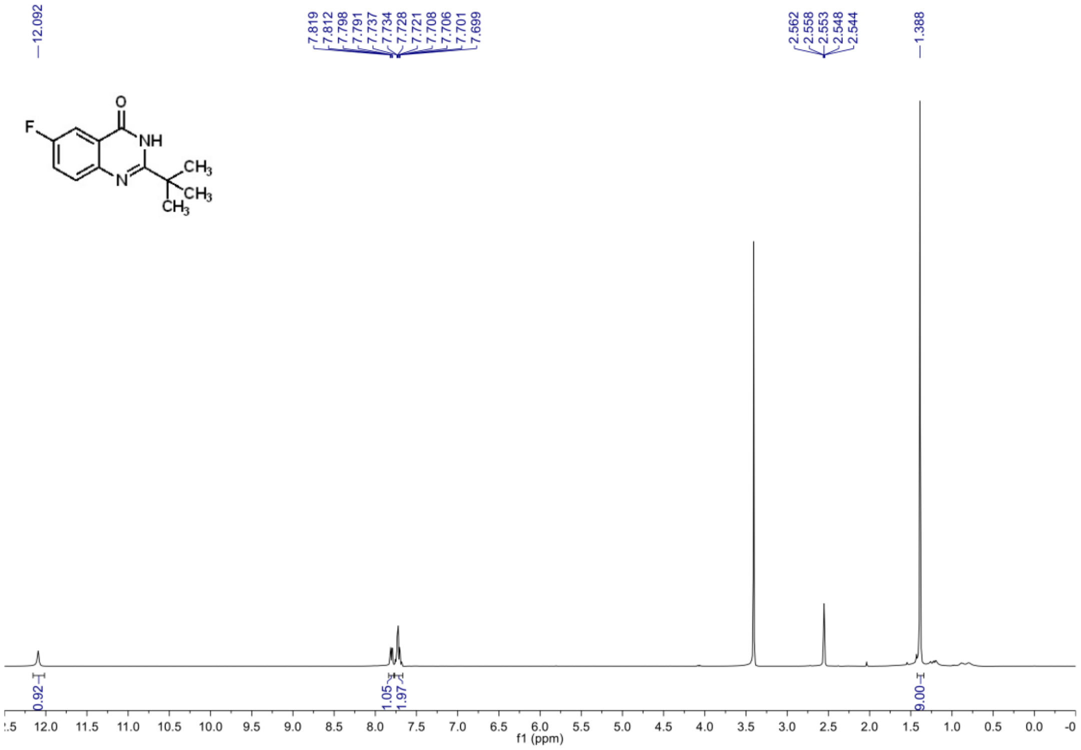

^{1}H NMR (400 MHz, DMSO-d_6) of compound **3l.**

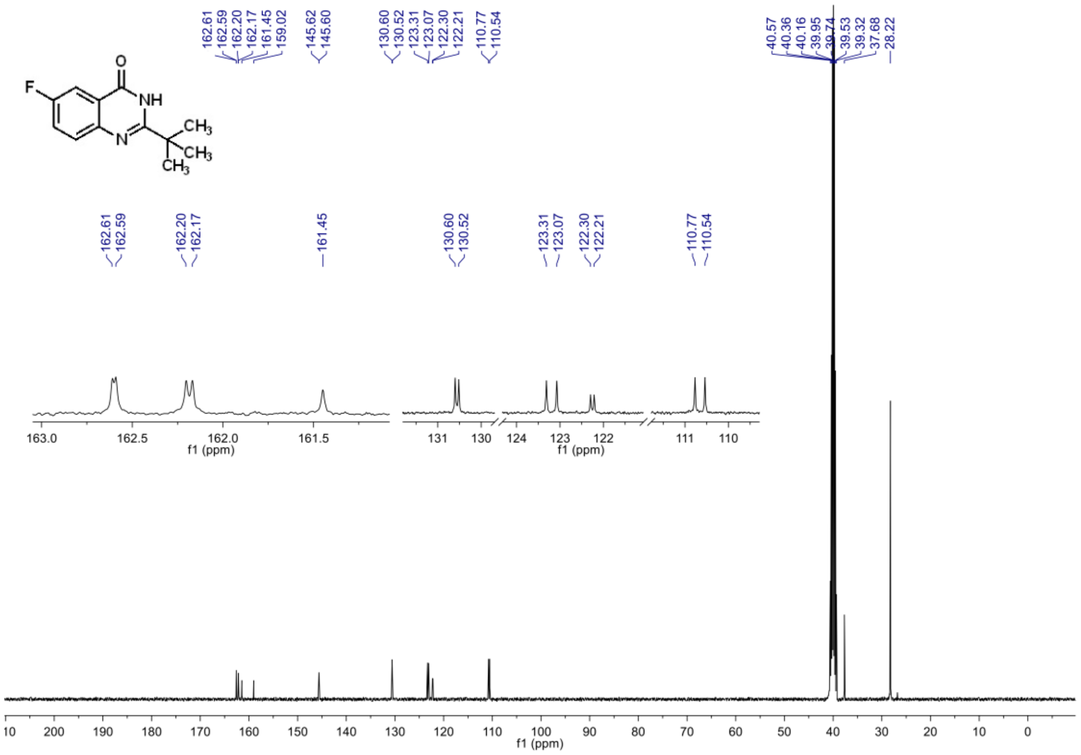

^{13}C NMR (100 MHz, DMSO-d_6) of compound **3l.**

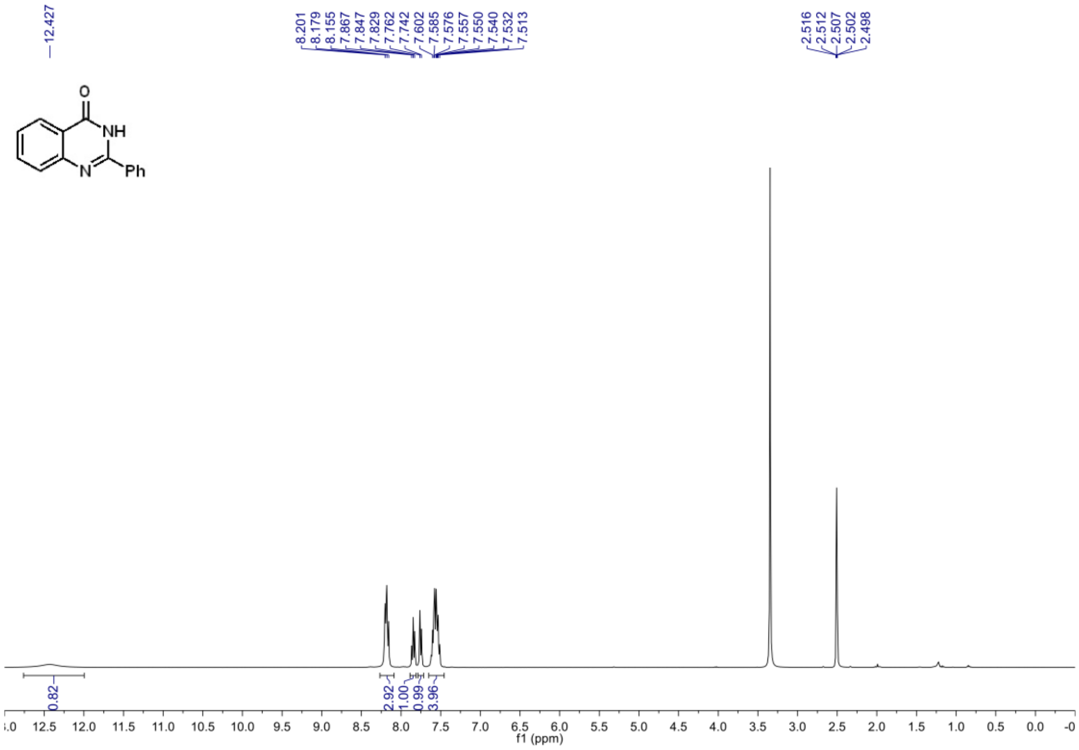

^{1}H NMR (400 MHz, DMSO-d_6) of compound **3m.**

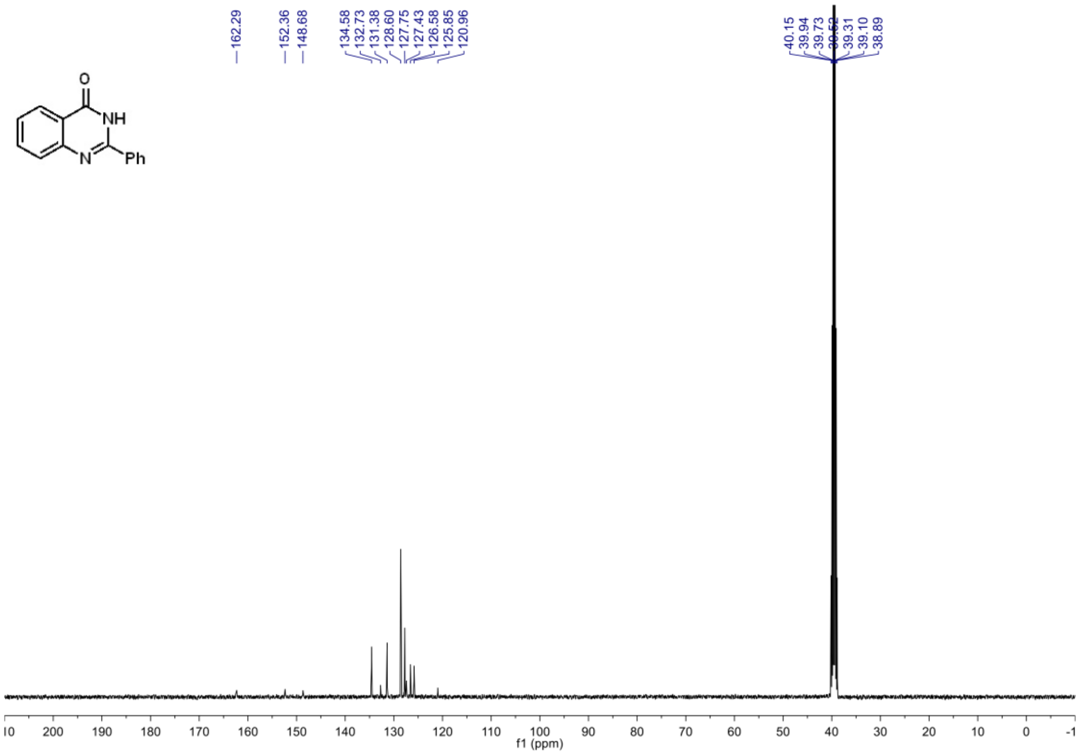

^{13}C NMR (100 MHz, DMSO-d_6) of compound **3m.**

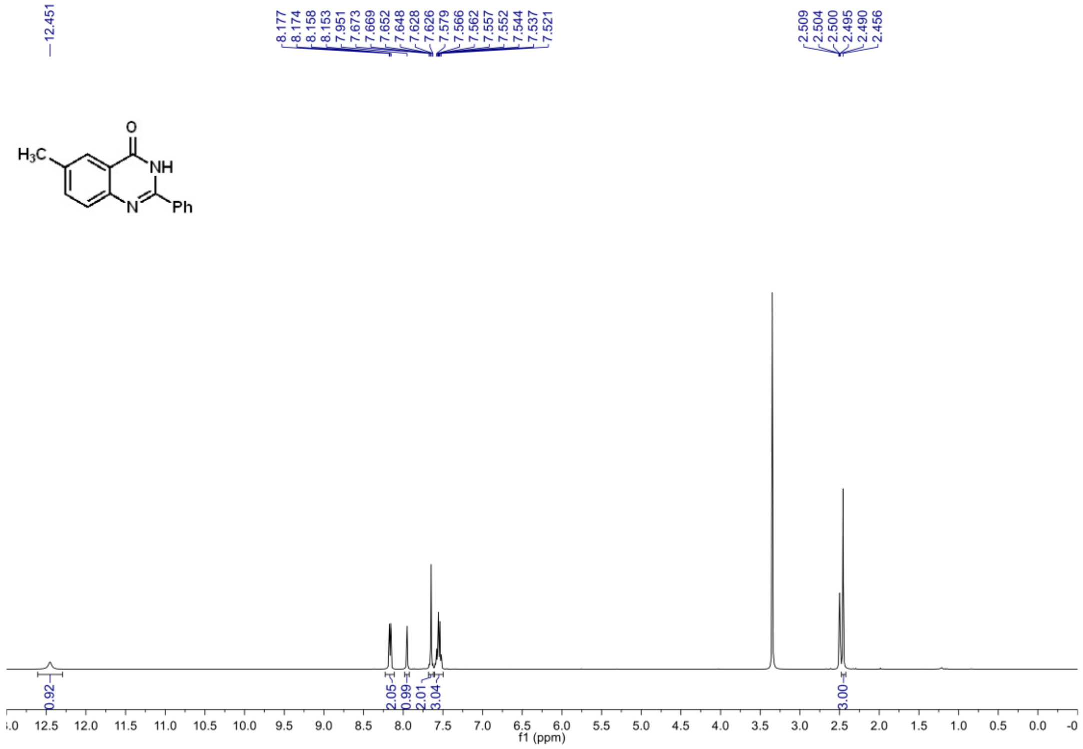

^{1}H NMR (400 MHz, DMSO-*d6*) of compound **3n.**

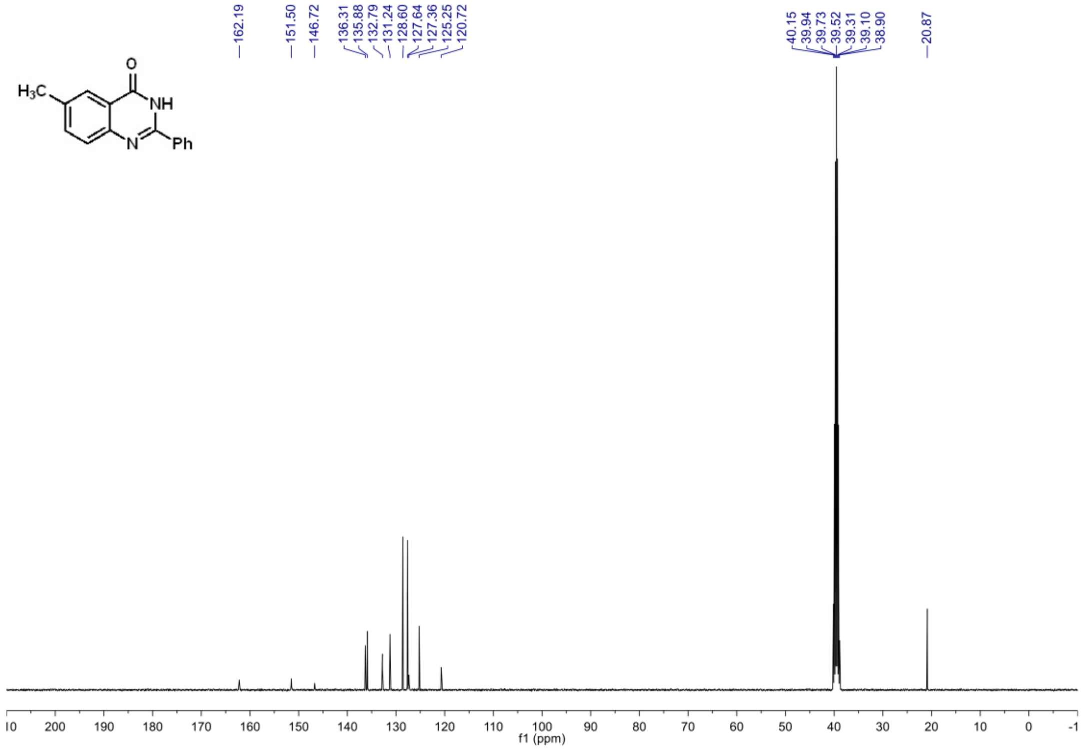

^{13}C NMR (100 MHz, DMSO-*d6*) of compound **3n.**

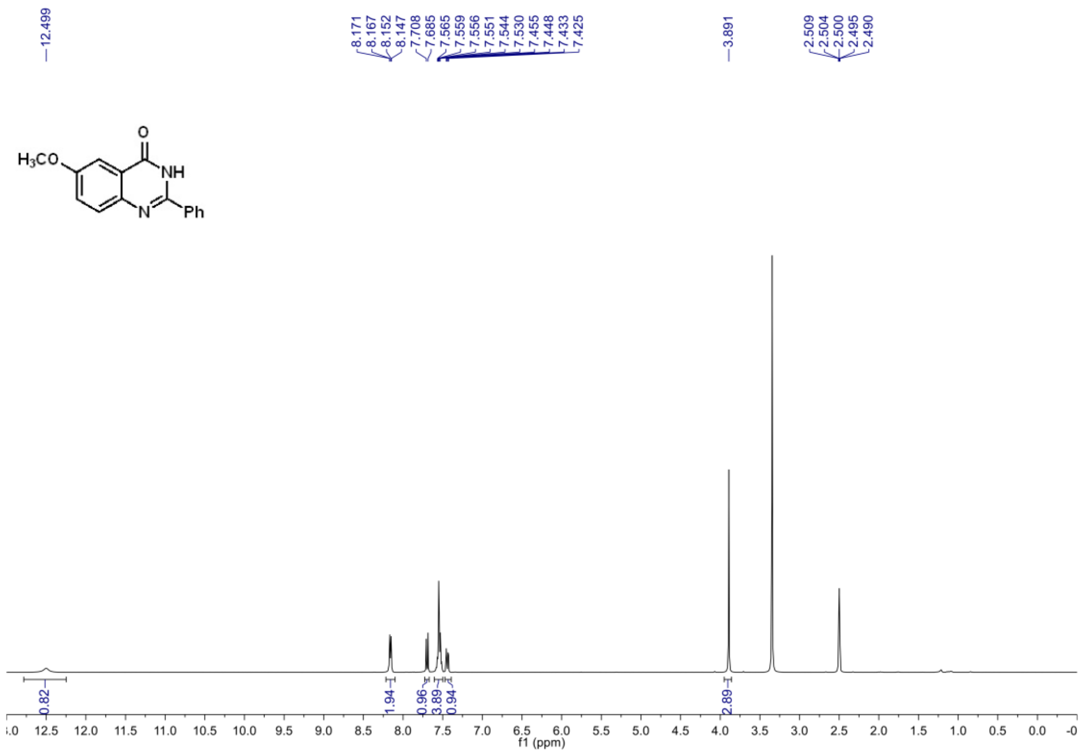

^{1}H NMR (400 MHz, DMSO-d_6) of compound **3o.**

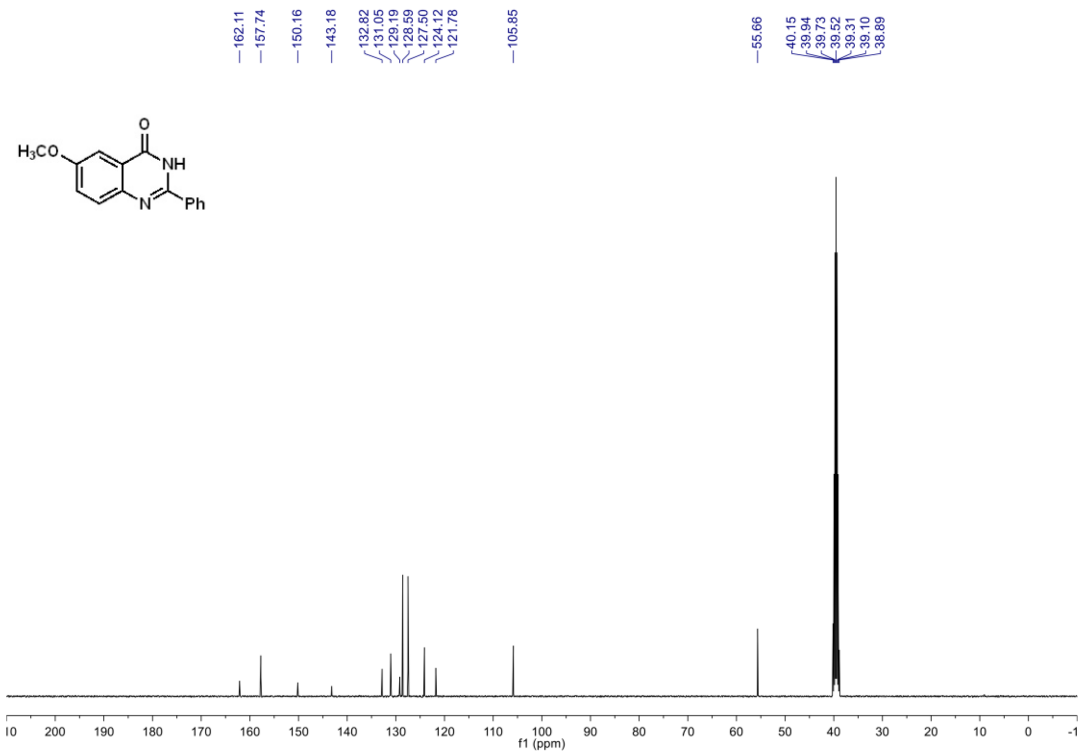

^{13}C NMR (100 MHz, DMSO-d_6) of compound **3o.**

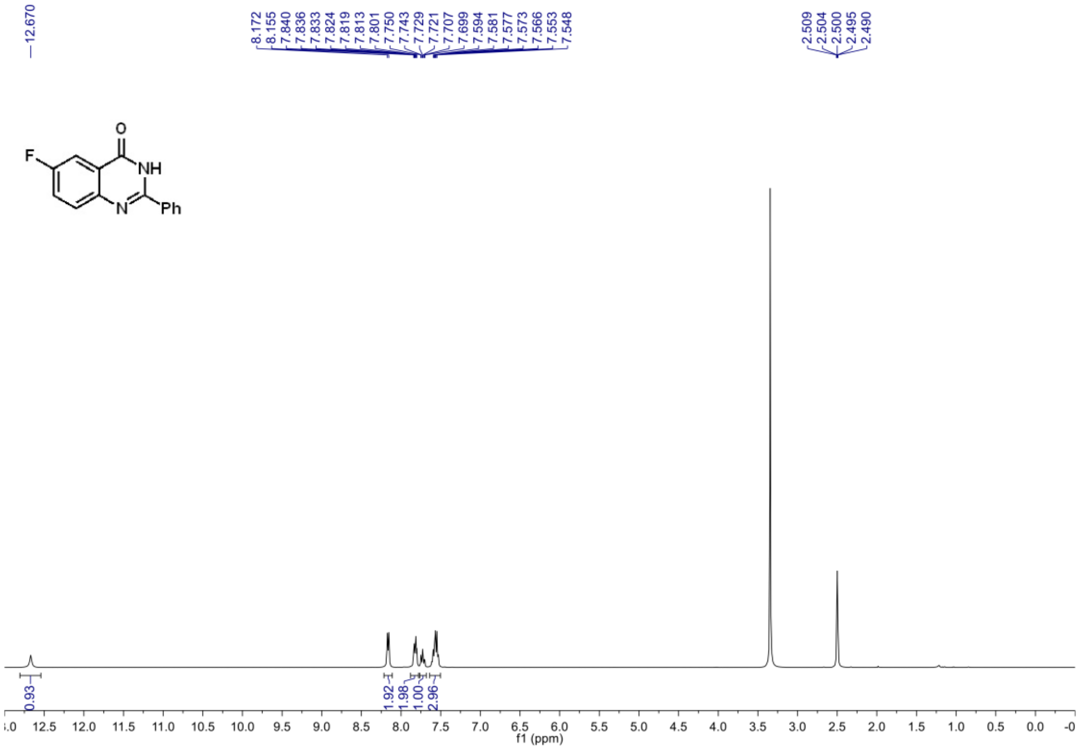

^{1}H NMR (400 MHz, DMSO-d_6) of compound **3p.**

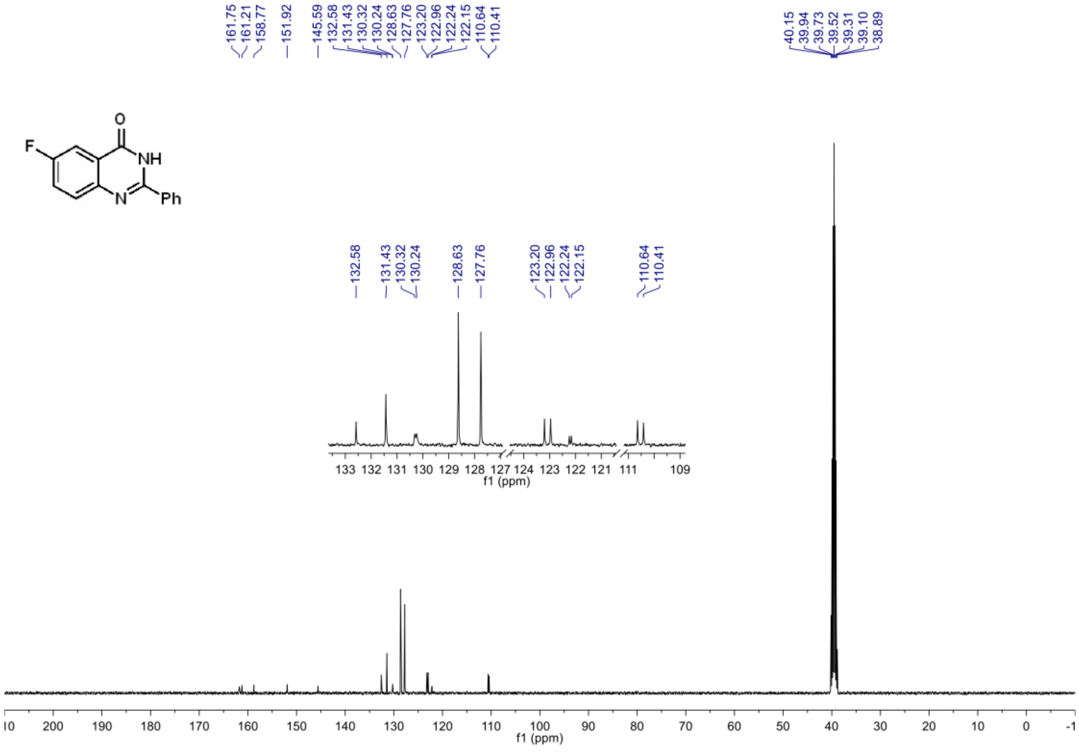

^{13}C NMR (100 MHz, DMSO-d_6) of compound **3p.**
